# Air quality and health co-benefits of China’s carbon dioxide emissions peaking before 2030

**DOI:** 10.1038/s41467-022-28672-3

**Published:** 2022-02-23

**Authors:** Rong Tang, Jing Zhao, Yifan Liu, Xin Huang, Yanxu Zhang, Derong Zhou, Aijun Ding, Chris P. Nielsen, Haikun Wang

**Affiliations:** 1 Joint International Research Laboratory of Atmospheric and Earth System Sciences, School of Atmospheric Sciences, Nanjing University, Nanjing, 210023 China; 2 State Key Laboratory of Pollution Control and Resource Reuse, School of Environment, Nanjing University, Nanjing, 210023 China; 3 State Environmental Protection Key Laboratory of Environmental Planning and Policy Simulation, Chinese Academy of Environmental Planning, Beijing, 100012 China; 4 Collaborative Innovation Center of Climate Change, Jiangsu Province, Nanjing, 210023 China; 5 Frontiers Science Center for Critical Earth Material Cycling, Nanjing University, Nanjing, 210023 PR China; 6 Harvard-China Project on Energy, Economy and Environment, Harvard John A. Paulson School of Engineering and Applied Sciences, Harvard University, Cambridge, MA 02138 USA

**Keywords:** Climate-change mitigation, Environmental impact, Sustainability, Developing world

## Abstract

Recent evidence shows that carbon emissions in China are likely to peak ahead of 2030. However, the social and economic impacts of such an early carbon peak have rarely been assessed. Here we focus on the economic costs and health benefits of different carbon mitigation pathways, considering both possible socio-economic futures and varying ambitions of climate policies. We find that an early peak before 2030 in line with the 1.5 °C target could avoid ~118,000 and ~614,000 PM_2.5_ attributable deaths under the Shared Socioeconomic Pathway 1, in 2030 and 2050, respectively. Under the 2 °C target, carbon mitigation costs could be more than offset by health co-benefits in 2050, bringing a net benefit of $393–$3,017 billion (in 2017 USD value). This study not only provides insight into potential health benefits of an early peak in China, but also suggests that similar benefits may result from more ambitious climate targets in other countries.

## Abstract (web summary)

Understanding benefits of carbon mitigation is an important impetus for governments to adopt more ambitious climate targets. Here, the authors show positive air quality and health co-benefits are possible if China’s CO_2_ emissions peak before 2030.

## Introduction

According to the Intergovernmental Panel on Climate Change (IPCC) 1.5 °C Special Report, the rise in global average surface temperatures could reach 1.5 °C between 2030 and 2052, posing great threats to human and natural systems^1^. Failure to act on climate change would result in huge socio-economic losses, potentially far outweighing the mitigation costs^2^. It is thus urgent to implement climate policies to curb the continuous rise in emissions of greenhouse gases (GHGs). As the largest carbon emitter in the world, China recently vowed to peak its carbon dioxide emissions before 2030 and achieve carbon neutrality before 2060, strengthening the earlier commitments of its nationally determined contribution (NDC) under the Paris Agreement to peak carbon “around” 2030. The carbon neutrality pledge also signaled that China is scaling up the ambition of its NDC as agreed in Paris.

Given many shared anthropogenic sources of GHGs and air pollutants, mitigation policies aimed at CO_2_ reductions could bring health co-benefits from abated air pollution^3,4^. At a global level, the costs of reducing GHGs emissions could be more than offset by the health co-benefits by 2050^5^. Yet the cost-benefit ratios of achieving NDC targets differ between developed and developing countries. Developing countries like India and China would gain considerable health benefits via air pollution abatement, up to 3–9 times the implementation costs in 2050^6^, whereas the comparable ratios in the European Union and USA would be relatively modest, at 7–84% and 10–41%, respectively^5^.

Notably, China’s carbon emissions peak is now estimated to occur ahead of 2030, the year of its NDC target, owing to structural economic changes and enhanced emission reduction actions. As projected in our previous research, emissions for China may peak before 2025, at least 5 years ahead of China’s Paris target of 2030^7^, and other recent studies have likewise shown realistic paths to surpass the NDC target^8,9^. With the peak year moving forward significantly, existing literature based on China’s NDC commitment likely overestimates its projected cumulative CO_2_ emissions and underestimates related co-benefits in air quality and public health^10^. Investigation into the social and economic impacts of such an early carbon peak in China, moreover, is still scarce. Besides, consideration of the diversity of climate change paths remains insufficient in the existing studies on the synergistic impact of climate policy on air quality^11^.

**Fig. 1:**
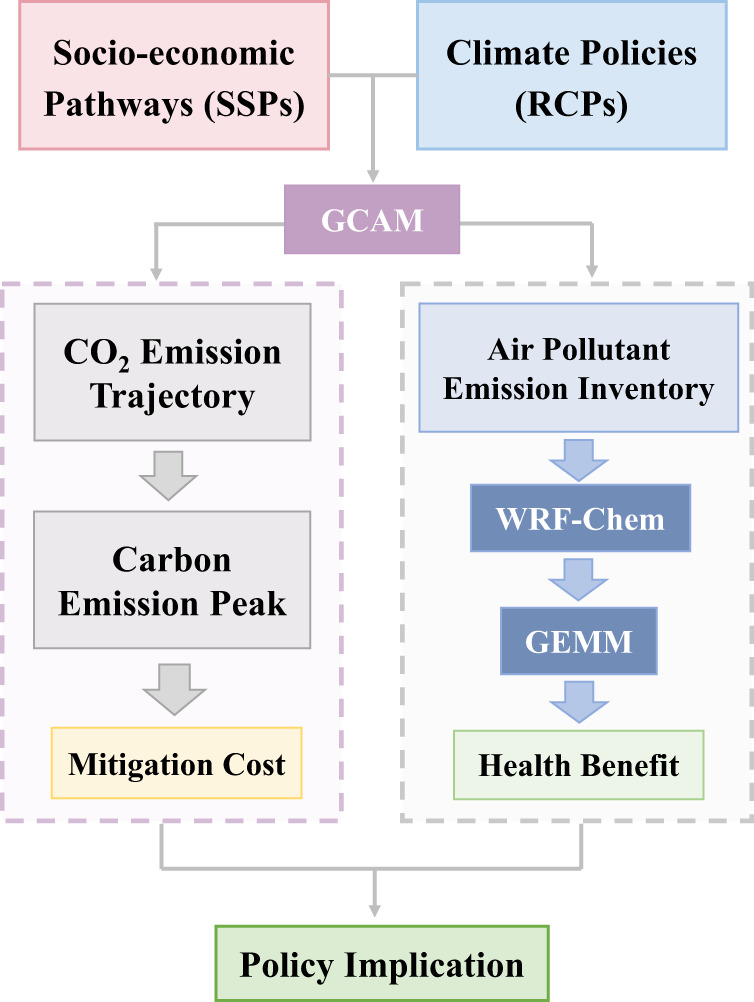
Methodological framework of this study. An integrated assessment framework is applied to assess the air quality and health co-benefits of China’s early carbon emission peak. Scenarios in this study are based on combinations of the SSPs and RCPs. The GCAM model is used to estimate CO_2_ and air pollutant emissions under each scenario. The ambient PM_2.5_ concentrations and related mortalities under each scenario are then quantified by combining the WRF-Chem and GEMM model. Finally, the health benefits are monetized using the value of statistical life (VSL) and compared to the costs of implementing climate policies.

This study, a comprehensive assessment of air quality and health co-benefits of climate policies in China, applies an integrated assessment framework (Fig. 1, see details in the “Methods”) to estimate the air quality and health co-benefits under different carbon mitigation pathways. We utilize chemical transport modeling and health risk assessment to quantify the cooperative effects of climate polices on ambient PM_2.5_ concentrations and the mortality from long-term PM_2.5_ exposure. We find that implementation of climate policies in China would reduce PM_2.5_-related mortality and yield huge health co-benefits, especially in the long run. The more stringent the climate policy is, the greater the health benefits it would bring. The health co-benefits could fully offset the carbon mitigation costs in the long-term, to 2050. However, the co-benefits of climate policies alone will not be enough to realize China’s air quality goals and prevent the growth of the PM_2.5_-related health burden before 2050, especially given the increasing population aging.

## Results and discussion

### CO_2_ emission trajectory

Nine scenarios combining the characters of socio-economic developments and the ambitions of climate policies are considered in this study, represented respectively by the Shared Socioeconomic Pathways (SSPs) and Representative Concentration Pathways (RCPs). When a specific long-term radiation forcing (RF) target (e.g., 2.6 W/m^2^ in 2100 under RCP2.6) is imposed as a quantitative constraint on the SSPs (e.g., SSP1, SSP2 or SSP5), the cumulative carbon emissions by 2100 are fixed to meet that RF level for various scenarios, labeled accordingly as SSP1_RCP2.6, SSP2_RCP2.6, and SSP5_RCP2.6 in Table 1. The set of climate policy assumptions (SSPx_RCPx) will thus determine the outcomes of the scenario analysis, including on energy structure and carbon trajectories (see Supplementary Note 1.2).

**Table 1:** Description of scenario settings.

Scenarios	Soci-economic pathways			Radiative forcing level by 2100
	Description	Challenges to mitigation	Challenges to adaptation	
SSP1_REF	Sustainable development	Low	Low	–
SSP1_RCP2.6				2.6 W/m^2^
SSP1_RCP1.9				1.9 W/m^2^
SSP2_REF	Middle of the road	Medium	Medium	–
SSP2_RCP2.6				2.6 W/m^2^
SSP2_RCP1.9				1.9 W/m^2^
SSP5_REF	Fossil-fueled development	High	Low	–
SSP5_RCP2.6				2.6 W/m^2^
SSP5_RCP1.9				1.9 W/m^2^

In China, industry and power generation are the two major sources of CO_2_ emissions^12^, contributing more than 80% of the total emissions in 2010 due to the sectors’ intensive use of fossil fuels, especially coal (Supplementary Table 2). Adjustment of the energy structure is the key to mitigating China’s CO_2_ emissions. For example, to achieve the 1.5 °C goal under the SSP1 pathway (i.e., SSP1_RCP1.9), the fossil fuel share of total primary energy would decline from 91.6% under SSP1_REF to 83.8% under SSP1_RCP1.9 in 2030, and from 85.2% to 42.9% in 2060 (Supplementary Fig. 2 and Note 1.3). And the share of biomass fuels and non-biomass renewable energy (e.g., solar and wind) under SSP1_RCP1.9 would respectively increase to 24.1% and 31.4% in 2060. A strong upscaling of non-fossil fuels (e.g., biomass fuels and nuclear power) also occurs in the SSP2_RCP1.9 and SSP5_RCP1.9 scenarios. Moreover, significant deployment of carbon capture and storage (CCS) seems to be an essential step, especially to achieve more ambitious climate targets in the future. For the SSP2 and SSP5 scenarios, most CO_2_ emissions from fossil fuels (~98%) and biomass fuels (~92%) used in electricity generation and industry would be captured and stored by 2050 (Supplementary Fig. 3).

**Fig. 2:**
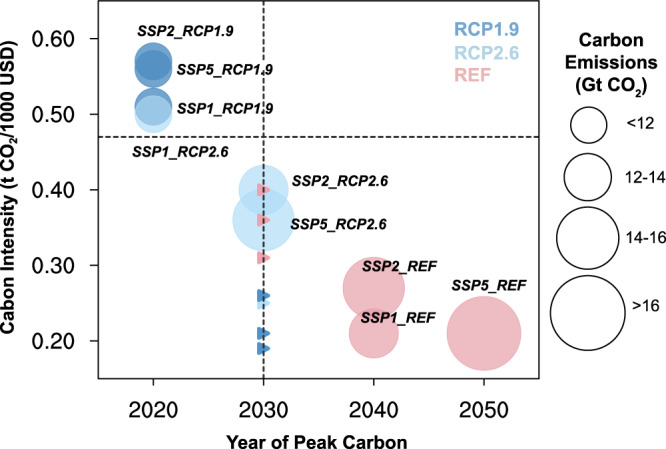
Years of peak carbon emissions and the carbon intensity in both peak years and 2030 for all nine scenarios. The size of the circles represents the total carbon emissions, and their centers correspond to the year of peak carbon and the value of carbon intensity in that year. Note that the model results of peak years from GCAM here are at 10-year intervals. REF, RCP2.6, and RCP1.9 are marked in pink, light blue and blue, respectively. The two dashed lines represent China’s NDC targets of peak year of 2030 and carbon intensity by 2030 (i.e., 0.47t-CO_2_/1000 USD). The carbon intensity in 2030 for the 7 scenarios peaking earlier or later than that year are marked by triangles.

The rapid shift away from fossil fuels towards zero- or low-carbon energy sources and increased use of CCS under a strict climate target would lead to substantial reduction in carbon emissions. As illustrated in Fig. 2, under all analyzed mitigation scenarios (i.e., RCP2.6 and RCP1.9), China’s CO_2_ emissions would peak at 10,423–15,500 Mt CO_2_ yr^−1^ in or before 2030, meeting the current NDC target of “around 2030” (Supplementary Table 1). Under the 1.5 °C scenarios (RCP1.9), the peak would occur earlier, around 2020 (10,645~11,901 Mt CO_2_ yr^−1^), meeting China’s newer peak year commitment of “before 2030.” Note also that China would be projected to achieve another NDC commitment of cutting CO_2_ emissions per unit gross domestic product (GDP) to 0.47 t-CO_2_/1000 USD, a reduction of 60% from 2005 levels, by 2030 in all scenarios (see Fig. 2 and Supplementary Fig. 1).

### Co-benefits for ambient air pollution

**Fig. 3:**
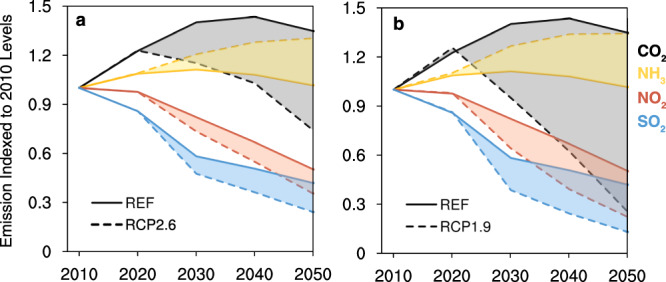
Emission trends of CO_2_, NH_3_, NO_X_, and SO_2_ under various scenarios in China through 2010–2050. **a** Comparison of emissions between SSP1_REF and SSP1_RCP2.6 indexed to 2010 levels. The solid lines represent the REF, while the dashed lines represent RCPs. **b** is the same as (**a**), but for the comparison between SSP1_REF and SSP1_RCP1.9 instead.

The implementation of climate policies to mitigate CO_2_ emissions would yield co-benefits in reduced air pollutant emissions in China (Fig. 3, Supplementary Figs. 4, 5). Emissions of air pollutants, with the exception of ammonia (NH_3_), decrease with reductions of CO_2_ emissions as the climate policy becomes more stringent. But the level of mitigation differs by pollutant species, mainly reflecting their specific sectoral sources (Fig. 3). For example, as energy use is the primary source of sulfur dioxide (SO_2_), nitrogen oxides (NO_X_) and CO_2_ emissions^12^, CO_2_ mitigation measures such as reduced coal consumption bring great co-reductions in SO_2_ and NO_X_ emissions. As the major sources of volatile organic compounds (VOCs), black carbon (BC) and organic carbon (OC) emissions do not overlap with major sources of CO_2_ (see Supplementary Table 3 and Table 5), it makes sense that the co-benefits of CO_2_ reductions on their emissions are not as significant as those of SO_2_ and NO_X_. In China, land use (including agriculture) produces almost all (>99%) of NH_3_ emissions, which are therefore barely affected by policies to mitigate CO_2_ in the scenarios. In fact, separate from the growing food demand that accompanies economic development, large amounts of biomass fuels are required in all climate policy scenarios (both RCP2.6 and RCP1.9) (Supplementary Fig. 2 and Note 3.1); both lead to increased ammonia emissions due to increased demand for and application of nitrogenous fertilizer (Fig. 3).

**Fig. 4:**
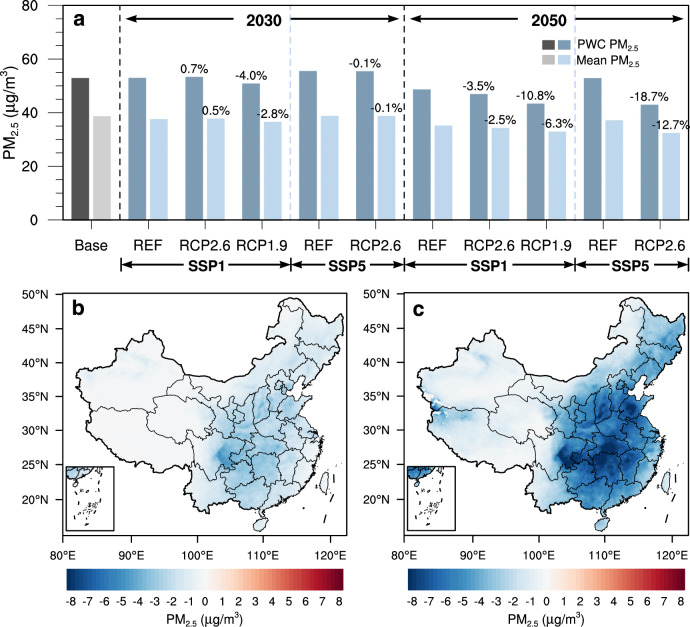
Changes in PM_2.5_ concentrations brought about by climate policies under various scenarios. **a** PWC-PM_2.5_ and annual mean PM_2.5_ concentrations under different scenarios in 2030 and 2050. Fractional changes (%) relative to the corresponding REF scenarios are noted; **b**, **c** Spatial distribution of PM_2.5_ changes between SSP1_RCP1.9 and SSP1_REF in 2030 and 2050, respectively. Note that 2015 is chosen as the base year.

In 2015, the population-weighted concentration of PM_2.5_ (PWC-PM_2.5_) was 53.0 μg/m^3^ in China. The implementation of different climate policies will result in different levels of PM_2.5_ mitigation relative to the REF scenario (see Fig. 4a, Supplementary Table 6 and Fig. 7), and the climate policies will yield greater improvements to air quality in the long run, as they become more stringent. For example, the PWC-PM_2.5_ in the SSP1_RCP1.9 scenario is 50.9 μg/m^3^ in 2030 and 2.1 μg/m^3^ lower than that of the SSP1_REF scenario (Fig. 4b), while in 2050 it will decrease to 43.4 μg/m^3^ and 5.1 μg/m^3^ lower (Fig. 4c). Under the same socio-economic path (SSP), the RCP1.9 scenario has a lower PWC-PM_2.5_ than the RCP2.6 scenario. This implies that stricter climate policies would not only mitigate more CO_2_ emissions but also lead to greater air quality improvement. In 2050, SSP1_RCP1.9’s PWC-PM_2.5_ is 3.5 μg/m^3^ lower than that in the RCP2.6 scenario.

Nevertheless, the co-benefits of climate policies alone on air pollution mitigation are limited. With the most ambitious assumptions of the SSP1_RCP1.9 scenario, about 778 million people in China will still be exposed to ambient air with annual average PM_2.5_ concentrations above 35 μg/m^3^ in 2050. This will not meet the latest air quality objective declared in the country’s “Beautiful China” strategy, which aims to reduce PM_2.5_ concentrations in all Chinese cities to at most 35 μg/m^3^ by 2035. The co-benefits of climate policies will not be enough to realize China’s air quality goals, which will also require strengthening of dedicated air pollution control policies^10^.

It is notable that the PWC-PM_2.5_ of SSP1_RCP2.6 is slightly higher (0.4 μg/m^3^) than that of SSP1_REF in 2030 (Supplementary Fig. 8a). Especially for provinces with large agricultural outputs like Henan, Shandong, and Jiangsu^13^, their PWC-PM_2.5_ levels in the SSP1_RCP2.6_2030 scenario are higher than those in the SSP1_REF_2030 scenario, by 0.7, 0.5, and 0.5 μg/m^3^, respectively in these three provinces (see Supplementary Note 3.1). Because emissions of the acidic gases NO_X_ and SO_2_ do not decline enough by 2030 under SSP1_RCP2.6, increasing NH_3_ emissions (Fig. 3) discussed above may hinder PM_2.5_ mitigation by enhancing formation of ammonium nitrate and sulfate^14,15^. These key forms of secondary PM_2.5_ comprise nearly 30% of China’s annual average concentration of PM_2.5_^16,17^. See details in Supplementary Note 3.

### Health benefits

**Fig. 5:**
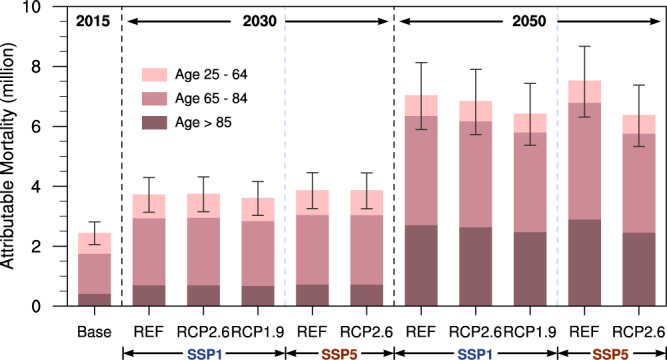
Age-specific mortality attributable to PM_2.5_ exposure estimated by GEMM_NCD+LRI in 2015, 2030, and 2050 under various SSP_RCP scenarios in China. Data are presented as “mean values” of mortality with uncertain intervals based on the 95% CIs of RR. Note that relative risk in GEMM was applied for adults over 25 years old.

In 2015, the deaths attributable to ambient PM_2.5_ exposures in China are estimated at 2,441,900 (95% CI, 2,052,000–2,809,100), recognizing that in reality the mortality impacts of chronic exposure to air pollution may be distributed over years or even decades after exposure^18,19^. In 2030, they grow to 3.6–3.9 million across all scenarios and increase to 6.4–7.5 million in 2050, with the elderly (>65 yr) particularly at risk (see Fig. 5, Supplementary Table 7). We find that aging of the population is the leading causal factor of such growth (see Supplementary Note 4), similar to previous studies^20^. This reinforces that relying on the co-benefits of climate policy alone will be insufficient to prevent the growth of the PM_2.5_-related health burden in China before 2050, especially given aging of the population.

**Fig. 6:**
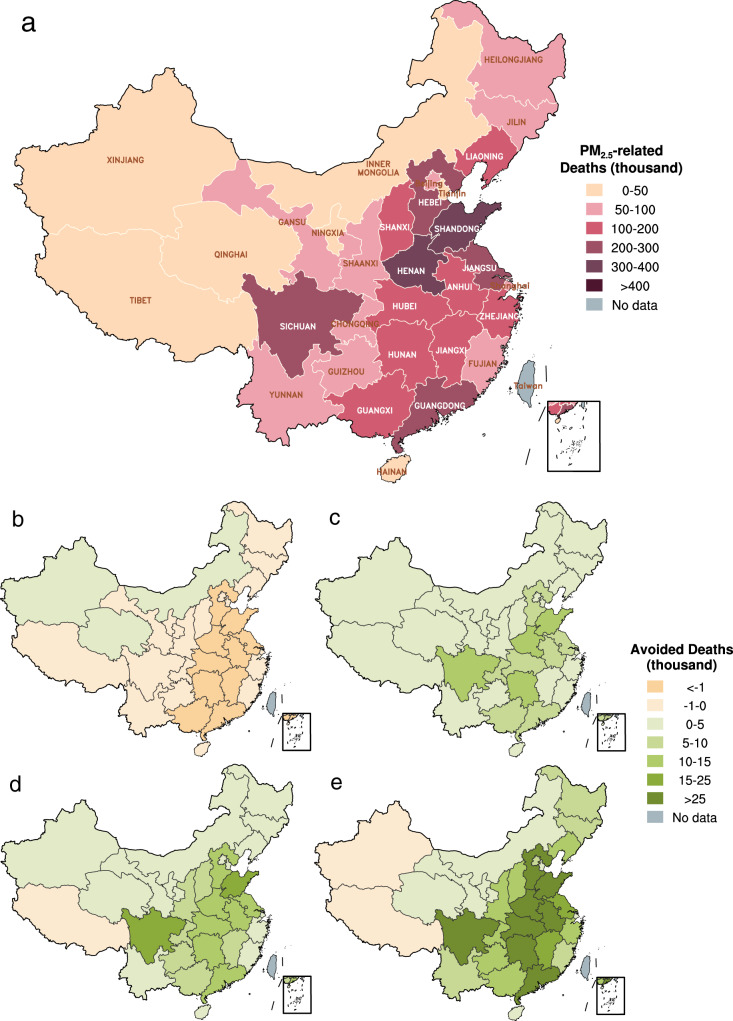
China’s PM_2.5_-attributable mortality and that prevented by climate policies under the SSP1 pathway. **a** PM_2.5_-attributable mortality in the SSP1_REF scenario in 2030. **b**, **c** Difference of PM_2.5_-attributable mortality with different climate mitigation targets under the SSP1 pathway in 2030: REF-RCP2.6 and RCP2.6-RCP1.9, respectively. **d**, **e** the same as (**b**, **c**) but in 2050.

Nevertheless, implementation of climate policies would yield considerable health co-benefits by reducing PM_2.5_-related mortality across all provinces in the analysis years of 2030 and 2050 (Fig. 6, Supplementary Table 8 and Fig. 9) compared to scenarios with no climate policy (SSPx_REF), except for SSP1_RCP2.6 in 2030 as discussed in the last section. And the more stringent the climate policy is, the greater the health benefits it would bring. For instance, the PM_2.5_-related deaths in SSP1_RCP1.9 are 3.7% and 6.1% lower than those in SSP1_RCP2.6 by 2030 and 2050, respectively (Fig. 6c and e). In addition, the health co-benefits of climate policies tend to increase in the longer run. For example, the PM_2.5_-related deaths in SSP1_RCP1.9 are 3.2% (118,000 deaths) and 8.7% (614,000 deaths) less than those in SSP1_REF, in 2030 and 2050, respectively (Fig. 6b and d). In addition to larger reductions in the long-run PM_2.5_ concentrations due to the reasons discussed above, sustained aging of the population exacerbates the baseline mortality risk of PM_2.5_ exposure (see Supplementary Note 4), both of which yield greater total reductions in PM_2.5_-related mortality in 2050 as a result of climate policies.

Applying estimates of mitigation costs and the value of statistical life (VSL) discussed in “Methods”, our analysis shows that the health co-benefits brought by CO_2_ emission reduction policies could fully offset the mitigation costs in the long-term, to 2050 (Table 2). Compared to the REF scenarios, SSP1_RCP2.6 and SSP5_RCP2.6 will produce net benefits of 393 billion and 3017 billion (in 2017 USD value) in 2050, respectively, equivalent to 0.45% and 2.77% of China’s GDP in 2050. Note that the mitigation cost mentioned here is from the Global Change Analysis Model (GCAM), which is an approximation of the welfare loss from emission mitigation efforts based on theoretical carbon tax revenue^21,22^. It refers to direct abatement costs such as those of new power plants and CCS technologies, but not the full macroeconomic costs in terms of GDP or consumption losses^23^. Co-reductions in air pollutant emissions under climate policies could also induce significant cost savings in pollution control, an additional co-benefit of climate policies that is not included in this study (see Supplementary Note 5.3).

**Table 2:** Comparison of costs and benefits of climate policies.

		Health benefit (billion USD)			Cost (billion USD)
		Local VSL*	Scaled International VSL*		
			β = 0.8	β = 0.4	
RCP2.6_2030	SSP1	−35.70	−343.48	−157.76	27.21
	SSP5	3.73	70.69	30.39	0
RCP2.6_2050	SSP1	561.19	7011.92	2222.12	167.91
	SSP5	3701.06	50,862.37	14,396.25	684.03
RCP1.9_2030	SSP1	205.01	979,615.31	449,936	—
RCP1.9_2050	SSP1	1737.87	12,298,345.65	3,897,418.53	—

*Note that the “Local VSL” and “Scaled International VSL” refers to monetized health benefits using the value of statistical life (VSL) from local surveys in China and those in foreign countries scaled to China, respectively, using approaches described in “Methods”.

### Policy implications

The peak of China’s CO_2_ emissions is almost certain to occur at some point during 2020–2030. China could realize its NDC commitment by peaking CO_2_ emissions around 2030 under the 2 °C scenarios, and earlier, before 2030, under the 1.5 °C scenarios. The stricter the climate policy and the cleaner the socio-economic pathway, the earlier the peak of CO_2_ emissions will occur and the larger the air quality and public health co-benefits achieved in the long run. Such health co-benefits could substantially if not fully offset the costs of more stringent climate policies and bring significant net economic benefits in 2050.

China’s Ministry of Ecology and Environment has set up a Department of Climate Change to promote synergies between climate change mitigation and air pollution control policies. Our results imply that the path of sustainable, low-carbon social and economic development (SSP1) will bring great environmental and health benefits for China at an affordable cost, especially in the long run. Implementing climate policies as soon as possible, especially decarbonizing the energy structure and applying CCS in industry and power sectors, can not only ensure achievement of the emission reduction target set in China’s NDC, but also bring more net co-benefits in air quality, public health and the economy. However, relying on the air pollution co-benefits of such climate policies alone is insufficient to prevent the growth of PM_2.5_-related mortality in China, and it is also necessary to adopt other measures, including further tightened conventional pollution controls, greening infrastructure with alternative building materials, and designing cities to encourage low-emission lifestyles.

A climate policy that increases the share of biomass fuels will likely combine with growing food demand to increase use of chemical fertilizer and, therefore, NH_3_ emissions, potentially exacerbating PM_2.5_ pollution and related health burdens in some regions. Measures should be taken to effectively control NH_3_ emissions. These could include enhancing the nitrogen use efficiency of fertilizer application and optimizing the scale of agricultural operations^24,25^, which may also reduce emissions of N_2_O, another greenhouse gas^26^. Informed by improved understanding of complex air pollution formation mechanisms (including emissions, atmospheric transport, and secondary chemistry), future policy analyses should also attach greater importance to both synergies and trade-offs between climate change mitigation and air pollution control. Only through policies that coordinate control of air pollution and carbon emissions can policymakers maximize their total benefits.

### Limitations

We used effects of PM_2.5_ exposures to estimate the total health burden caused by air pollution. While ignoring health effects of exposure to other air pollutants (e.g., O_3_ and NO_X_) may lead to underestimation, it is likely to be small because the health burden of PM_2.5_ is estimated to be around 6.2-fold greater than that of O_3_ on average^27^ and controversy exists about whether health effects of NO_X_ double-count those of nitrate PM_2.5_^28^. Also, the common approach of using mass concentrations of PM_2.5_ was used, disregarding composition, sources and particle size distribution. All of these characteristics may have different impacts on health^29^, but are commonly ignored in such studies as the epidemiological evidence is currently limited and inconsistent^30,31^.

In addition, uncertainties may be introduced by various factors in the integrated assessment model, GCAM, including methods of model application, selection of representative technologies and parameterization, and choice of the base year^32^. As we attempted to isolate the impact of climate policy on air pollution, we also did not consider special policies of future air quality control in our scenario analysis. For example, air quality could be further improved if stronger agricultural nitrogen management is implemented in the future, for example increasing the efficiency of nitrogen use during fertilizer application and agricultural waste management^24,33^. Nevertheless, the GCAM results for different scenarios here are reasonable, showing consistent trends in emissions of CO_2_ and air pollutants with the results of the other five integrated assessment models selected by the IPCC^23^.

Finally, since we mainly focus on the air pollution and health co-benefits of carbon reduction, the meteorological fields (from 2015) were assumed to remain the same for all scenarios and years, to eliminate possible confounding effects of climate change. Similarly, due to the lack of projected baseline mortality data for 2030 and 2050, we assume that the baseline mortality rates will remain the same as 2015 in the future.

## Methods

In this study, an integrated assessment framework was applied to investigate the economic costs and health benefits under different emission mitigation pathways in China (Fig. 1). Estimations of CO_2_ and air pollutant emissions under SSP-RCP scenarios were obtained from GCAM. Then, the ambient PM_2.5_ concentrations and related mortalities under each scenario were quantified by combining the Weather Research and Forecasting Model Coupled with Chemistry (WRF-Chem) and the Global Exposure Mortality Model (GEMM). Finally, the health benefits were monetized using VSL, and then compared to the costs of implementing the climate policies.

### Scenarios of future socio-economic status and emissions

In this paper, nine scenarios are selected across two dimensions (Table 1) to analyze the health co-benefits of climate polices in China. One dimension is socio-economic developments, i.e., the Shared Socioeconomic Pathways or SSPs^34^. The SSPs are defined along two different axes: challenges to climate mitigation and challenges to adaptation (see Supplementary Note 1.1). To investigate the effect of climate policies on air pollution, decarbonization paths for energy systems are the major focus in this study. We emphasize conditions resulting from different levels of climate mitigation. Additionally, to take account of the UN sustainable development goals and the general trend of globalization, SSPs with relatively low challenges to adaption (SSP1 and SSP5) but with different levels of climate mitigation challenges, i.e., SSP1 (low), SSP2 (medium) and SSP5 (high), are considered. SSP1 describes a world “taking the green road” with fewer adaptation and mitigation challenges, in which the population will peak in mid-century and then decline, while global per capita GDP will increase significantly. SSP5 describes a high-tech yet fossil-fuel-oriented world, with highly energy-intensive lifestyles. And SSP2 assumes a middle development path. The challenge of climate mitigation gradually increases in SSP1, SSP2, and SSP5.

The other dimension is the ambition of climate policies, using the Representative Concentration Pathways or RCPs. The REF assumes no policies explicitly designed to limit climate change. RCP2.6 and RCP1.9 assume paths limiting radiative forcing to 2.6 W/m^2^ and 1.9 W/m^2^ in 2100, exploring the possibility of limiting the rise in global mean surface temperature below 2 °C and 1.5 °C, respectively.

The SSP baseline scenarios (SSPx-REF) are developed with a set of qualitative assumptions given by the SSPs in the absence of climate polices. As the long-term radiation forcing target (from an RCP) is imposed on the SSP baselines as a quantitative constraint, the cumulative carbon emissions by 2100 are fixed (see Supplementary Note 1.2). This set of climate policy assumptions will have strong implications for the outcomes of the scenario analysis, including the energy structure and carbon trajectories. As above, the differences of air pollutant emissions between SSPx-REF and SSPx-RCPs reflect the synergistic effects of climate policies on air pollution control, such as accelerating clean energy deployment or constraints on land use change.

Here, we use the demographic and economic assumptions developed by KC and Lutz^35^ and Dellink et al.^36^, as shown in Supplementary Fig. 6. We also apply emission projections and potential mitigation costs from top-down simulations by GCAM in the selected SSP_RCP scenarios^37^. GCAM, an integrated assessment model, has been broadly applied in climate and energy assessments, including the latest IPCC Assessment Report^38^. The GCAM model searches for the equilibrium solution of carbon prices that cause all markets to be cleared and all consistency conditions set by the SSPs and RCPs narratives to be satisfied under the SSP_RCP framework at each time step. Thus, the emissions of air pollutants are determined by both pollutant emission factors set in the SSPs (see Supplementary Note 1.3) and the final energy consumption jointly determined by the environmental policy settings in SSP baselines and the level of radiation forcing defined by the RCPs^39^ (see Supplementary Note 1.2).

In general, carbon emission controls are applied to each activity and are strengthened as GDP rises, representing the historical trend that more stringent pollution control measures will be adopted as income levels increase^5^. The mitigation of non-CO_2_ GHGs is addressed by using parameterized functions for Marginal Abatement Cost (MAC) curves to change emission factors over time (see Supplementary Note 1.4). The mitigation cost from GCAM is an approximation of the welfare loss from emissions mitigation efforts by considering theoretical carbon tax revenue^21,22^. It can be thought of as the direct abatement cost (e.g., costs of new power plants, CCS technologies, etc.), but not the full macroeconomic costs in terms of GDP or consumption losses^23^. The model provides the mitigation costs of different energy and climate policies for each specific region.

### Simulation of surface PM_2.5_ concentration

According to the ambition of the landmark 2015 Paris Agreement to limit the increase in global average surface temperatures well below 2 °C, we compared the scenarios with or without climate policies seeking a maximum 2 °C temperature rise, i.e., RCP2.6 and REF, under the least and greatest socio-economic challenges to mitigation, i.e., SSP1 and SSP5, respectively. In addition, the SSP1_RCP1.9 scenario was added, in line with China’s raised recent ambitions and the stricter goal of 1.5 °C in the Paris Agreement. Thus, 11 parallel experiments were designed, including the base run in 2015 and 10 runs for 5 selected scenarios (i.e., SSP1_REF, SSP1_RCP2.6, SSP1_RCP1.9, SSP5_REF and SSP5_RCP2.6), in 2030 and 2050, respectively. Coupled dynamical and chemical simulations were conducted using WRF-Chem, version 3.6.1, and incorporated additional pathways of secondary pollution enhancement in China based on our previous studies^40^. The model has been demonstrated to reproduce pollution in China well and is widely used in previous studies, including our own^41,42^.

Consistent with previous studies, we selected the meteorological fields of a given year (2015) and conducted simulations of four representative months (January, April, July, October) for all scenarios^43^. The domain covers the Greater China region, including mainland China and part of East Asia, at a grid resolution of 20 km × 20 km. MIX, the mosaic Asian anthropogenic emission inventory developed by Tsinghua University^44^, was applied for the 2015 base run, while emissions in future scenarios were developed from GCAM model results^37^. The estimated biogenic inventory provided by MEGAN (the Model of Emissions of Gases and Aerosols from Nature) was also used^45^. The model configurations are given in detail in Supplementary Note 2.1.

We evaluated the simulated PM_2.5_ concentration against ground-based observations from almost 1500 stations (see Supplementary Note 2.3). Generally, our model well captures the seasonal variation and spatial distribution of PM_2.5_ concentration, with a Pearson correlation coefficient of ~0.81 at the annual average level (Supplementary Fig. 10). In addition, satellite-derived PM_2.5_ concentrations^46^ were used to further calibrate simulations and estimate PM_2.5_ exposures for all scenarios, similar to our previous study^47^.

### Estimation of the health impacts

Exposure to ambient air pollution, especially PM_2.5_, is a leading global health concern^48^. PM_2.5_ is currently the dominant contributor to mortality from long-term exposure to air pollution, adverse health impacts of which have been proved by extensive cohort epidemiological studies^49^. Thus, we focus on the health effects of long-term exposure to ambient PM_2.5_. We apply GEMM to estimate deaths attributable to ambient PM_2.5_ exposure for noncommunicable diseases (NCDs) and lower respiratory infections (LRIs) in China under the various scenarios^50^. The relative risk (RR) functions for GEMM were constructed on the basis of 41 cohort studies of outdoor air pollution exposures in 16 countries, including a recent cohort study in China^51^. The attributable-fraction type relationship presented in Eq. (1) was used to calculate the mortality attributable to outdoor PM_2.5_ exposure.

1 $${M}_{i,j}=\mathop{\sum}\limits_{g}{P}_{g,j} \, \times \, \hat{{I}_{i,j}} \, \times \, \left({{RR}}_{g,i,j}\left({C}_{g}\right)-1\right)$$

where

2 $$\hat{{I}_{i,j}}={I}_{i,j}/\overline {{{RR}}_{g,i,j}}$$

3 $$\overline{{{RR}}_{g,i,j}}=\frac{\mathop{\sum }\limits_{i=1}^{N}{P}_{g,j} \, \times \, {{RR}}_{g,i,j}\left({C}_{g}\right)}{\mathop{\sum }\limits_{i=1}^{N}{P}_{g,j}}$$

where, ${M}_{g,i,j}$ is the mortality and ${P}_{g,i}$ is the population of grid cell $g$ for disease *i* and age group *j*; ${I}_{i,j}$ is the reported national average annual disease (mortality) incidence rate for disease *i*, age group *j;* ${C}_{g}$ represents the PM_2.5_ concentration in cell $g$; ${{RR}}_{g,i,j}({C}_{g})$ is the relative risk at concentration ${C}_{g}$; $\overline{{{RR}}_{g,i,j}}$ represents the average population-weighted relative risk; $\hat{{I}_{i,j}}$ represents the hypothetical “underlying incidence” (cause-specific mortality rate) that would remain if PM_2.5_ concentrations were reduced to the theoretical minimum risk concentration (i.e., 2.4 μg/m^3^).

4 $${{RR}}_{g,i,\,j}\left({C}_{g}\right)=\left\{\begin{array}{cc}{{{{{\rm{exp }}}}}}\left\{\frac{{\theta }_{i,\,j}{{{{{\rm{log }}}}}}\left(\frac{{C}_{g}-2.4}{\propto }+1\right)}{1+{{{{{\rm{exp }}}}}}\left\{-\frac{{C}_{g}-2.4-{\mu }_{i,\,j}}{{v}_{i.j}}\right\}}\right\} & {if}{C}_{g} \, > \, 2.4\\ 1 & {{\mbox{otherwise}}}\end{array}\right.$$

where, 2.4 μg/m^3^ is the threshold concentration; $\theta$, $\alpha$, $\mu$ $v$ are fitted parameters of the concentration response functions for a given disease *j* provided by Burnett et al. (2018).

Here, we apply the disease incidence in 2015 derived from the Global Burden of Disease Results Tool 2017 version (GBD2017). For population size and spatial distribution, the LandScan Global Database (v2018) is adopted for 2015, and the projected population scenarios in SSPs are from NCAR’s IAM group and the City University of New York Institute for Demographic Research^52,53^. Age structure in 2015 and future scenarios are from GBD2017 and SSP database^35^, respectively.

The health effects of the climate policies are represented by differences ($\triangle {M}_{{{{{{{\rm{RCPs}}}}}}}}$) in the number of deaths attributable to air pollution between the RCP scenarios and their corresponding REF scenario, calculated by Eq. (5).

5 $$\triangle {M}_{{{{RCPs}}}}={M}_{{{{REF}}}}-{M}_{{{{RCPs}}}}$$

As the shape and parameters of the exposure–response (E–R) function are one the most important source of uncertainties in health impacts analysis^47^, we take the uncertainties in the E–R function as the uncertainties of estimated PM_2.5_-related mortality similar to previous studies^54,55^. Finally, the ~95% CI are calculated by inserting standard errors of GEMM parameters into the E–R function (see details in Supplementary Note 5.1).

In contrast with GEMM’s exclusive reliance on outdoor air pollution, the integrated exposure–response (IER) model adopted by the GBD incorporates risk information from both ambient PM_2.5_ pollution and other sources (e.g., secondhand smoke, household use of solid fuels). As suggested by a census-based epidemiological study, the GEMM results are better aligned with outcomes than the IER results in China^56^. The differences between effect estimates given by different relative risk functions (i.e., GEMM, IER2017) are analyzed in Supplementary Note 5.1.

### Valuation of the health benefits

To quantify the benefit of avoided mortality risks, we applied the Value of Statistical Life (VSL) to monetize the estimated health impacts. The VSL values in each scenario and year are calculated via Eq. (6),

6 $${{{\mbox{VSL}}}}_{{{\mbox{target}}}}={{{\mbox{VSL}}}}_{{{\mbox{baseline}}}}+({{{\mbox{INC}}}}_{{{\mbox{percap target}}}}-{{{\mbox{INC}}}}_{{{\mbox{percap baseline}}}})\times {{\mbox{MVSL}}}$$

where ${{{\mbox{VSL}}}}_{{{\mbox{baseline}}}}$ is the VSL value of a baseline year and MVSL is the adopted marginal VSL between the target and base years related to per capita disposable income (${{{\mbox{INC}}}}_{{{\mbox{percap}}}}$). And we adopt contingent valuation conducted in Chongqing which presented a linear relationship between VSL and income (i.e., VSL increased by 14,550 USD with annual income increases of 145.8 USD)^57,58^. Given the huge disparities between VSL values derived from different studies, even by one order of magnitude^55^, we calculated the VSL results in target years based several local studies in China for comparison. Moreover, since diverse VSL calculation methods may also lead to inconsistent results, we also calculated results on the basis of US EPA methodology^59^ in Supplementary Note 5.2. These results are much higher than the values calculated using local estimates from China. Considering that local estimates are more in line with local situation and more applicable, we opted to apply locally investigated results from Hammitt et al.^60^. See details in Supplementary Note 5.2.

### Reporting summary

Further information on research design is available in the Nature Research Reporting Summary linked to this article.

## Supplementary information

Supporting informaton

Reporting Summary

## Data Availability

The GCAM representation of the SSPs are supported by Calvin et al.^37^. The reanalysis data FNL can be downloaded from website: https://rda.ucar.edu/datasets/ds083.2/. The anthropogenic emission data MIX are available at website: http://meicmodel.org. The GEMM fit parameters are available at website: https://github.com/mszyszkowicz/DataGEMM. Baseline mortality information and age structure can be obtained using GBD Results Tool (version 2017) at website: https://gbd2017.healthdata.org/gbd-search/. Future age structure in SSP scenarios are available at website: https://tntcat.iiasa.ac.at/SspDb/. The LandScan population data are available at website: https://landscan.ornl.gov/. The future population size and spatial distribution for the SSPs can be obtained from website: https://www.cgd.ucar.edu/iam/modeling/spatial-population-scenarios.html. Additional data related to our results are available at figshare data publisher: https://figshare.com/articles/dataset/Data_rar/17648891 or on request from the corresponding authors.

## Code availability

The source code of the WRF-Chem model is archived on UCAR data repository (http://www2.mmm.ucar.edu/wrf/users/download). The code for assessing the PM_2.5_ health burden used in this study are available at website: https://github.com/evanliu3594/PM2.5-attr-mort). Data analysis and visualization in this study are mainly based on NCAR Command Language (NCL v6.4.1, https://www.ncl.ucar.edu/). Data processing techniques are available on request from the corresponding authors.
