# Supplementary material for: Air quality and health co-benefits of China’s carbon dioxide emissions peaking before 2030
Source: Nat Commun. 2022 Feb 23;13:1008. doi: 10.1038/s41467-022-28672-3 (PMC8866434; doi:10.1038/s41467-022-28672-3)
Supplement: Supplementary file 1 — Supporting informaton [file 41467_2022_28672_MOESM1_ESM.pdf]

Supplementary Information for

Air quality and health co-benefits of China's carbon dioxide emissions peaking before 2030

Rong Tang[a, #], Jing Zhao[b, c, #], Yifan Liu[b], Xin Huang[a, d], Yanxu Zhang[a], Derong Zhou[a, d], Aijun Ding[a, d, e], Chris P. Nielsen[f], Haikun Wang[a, d, e, *]

[a] Joint International Research Laboratory of Atmospheric and Earth System Sciences, School of Atmospheric Sciences, Nanjing University, Nanjing 210023, China
[b] State Key Laboratory of Pollution Control and Resource Reuse, School of Environment, Nanjing University, Nanjing 210023, China
[c] State Environmental Protection Key Laboratory of Environmental Planning and Policy Simulation, Chinese Academy of Environmental Planning, Beijing 100012, China
[d] Collaborative Innovation Center of Climate Change, Jiangsu Province, Nanjing 210023, China
[e] Frontiers Science Center for Critical Earth Material Cycling, Nanjing University, Nanjing, 210023, PR China
[f] Harvard-China Project on Energy, Economy and Environment, Harvard John A. Paulson School of Engineering and Applied Sciences, Harvard University, Cambridge, MA 02138, USA

# These authors contributed equally to this work.

* Correspondence to: Haikun Wang (wanghk@nju.edu.cn)

This Supplemental Information document presents the data, assumptions and methods of the primary document as well as an additional analysis.

This file includes:

Supplementary Figures
Supplementary Tables
Supplementary Note1: Mitigation pathways under SSP-RCP scenarios
Supplementary Note2: WRF-Chem simulations of ambient $PM_{2.5}$ in China
Supplementary Note3: Special case caused by ammonia emission
Supplementary Note4: Decomposition of changes in $PM_{2.5}$-related deaths
Supplementary Note5: Uncertainty analysis
References

Supplementary Figures

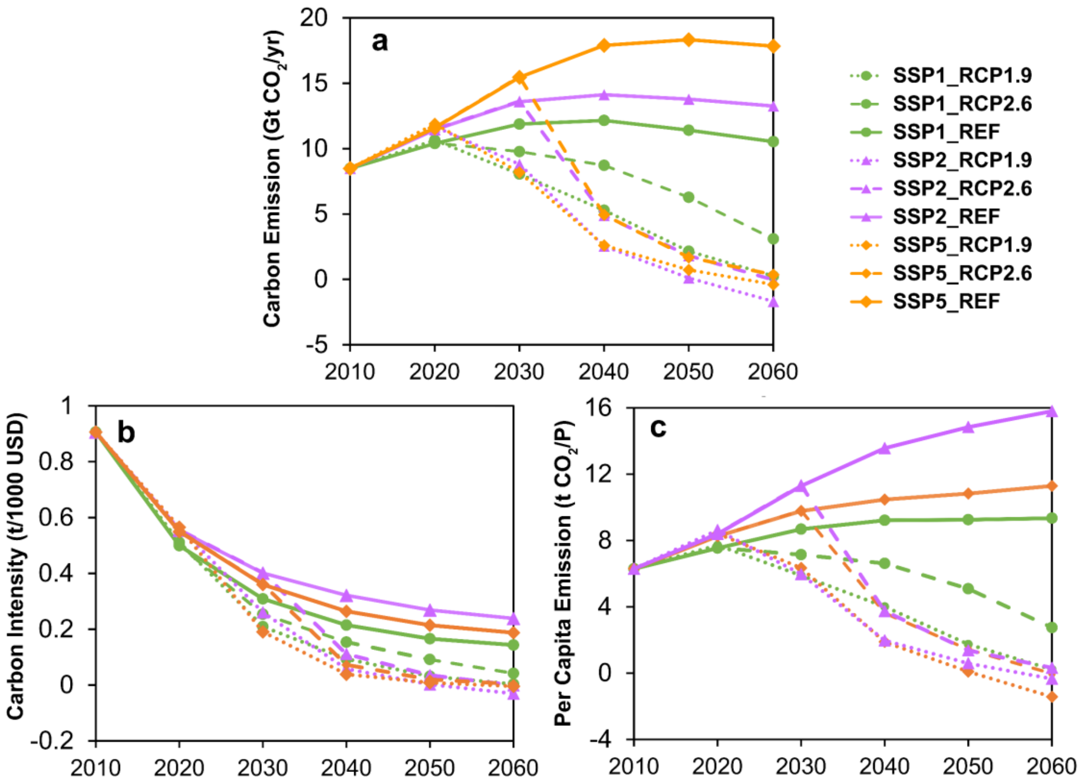

Supplementary Figure 1. Trajectories of China's carbon emissions under nine SSP_RCP scenarios from 2010 to 2060. a. Total carbon emissions, Mt CO_2/yr; **b.** Carbon emissions intensity, t-CO_2/1000 USD; **c.** Per capita carbon emissions, t CO_2.

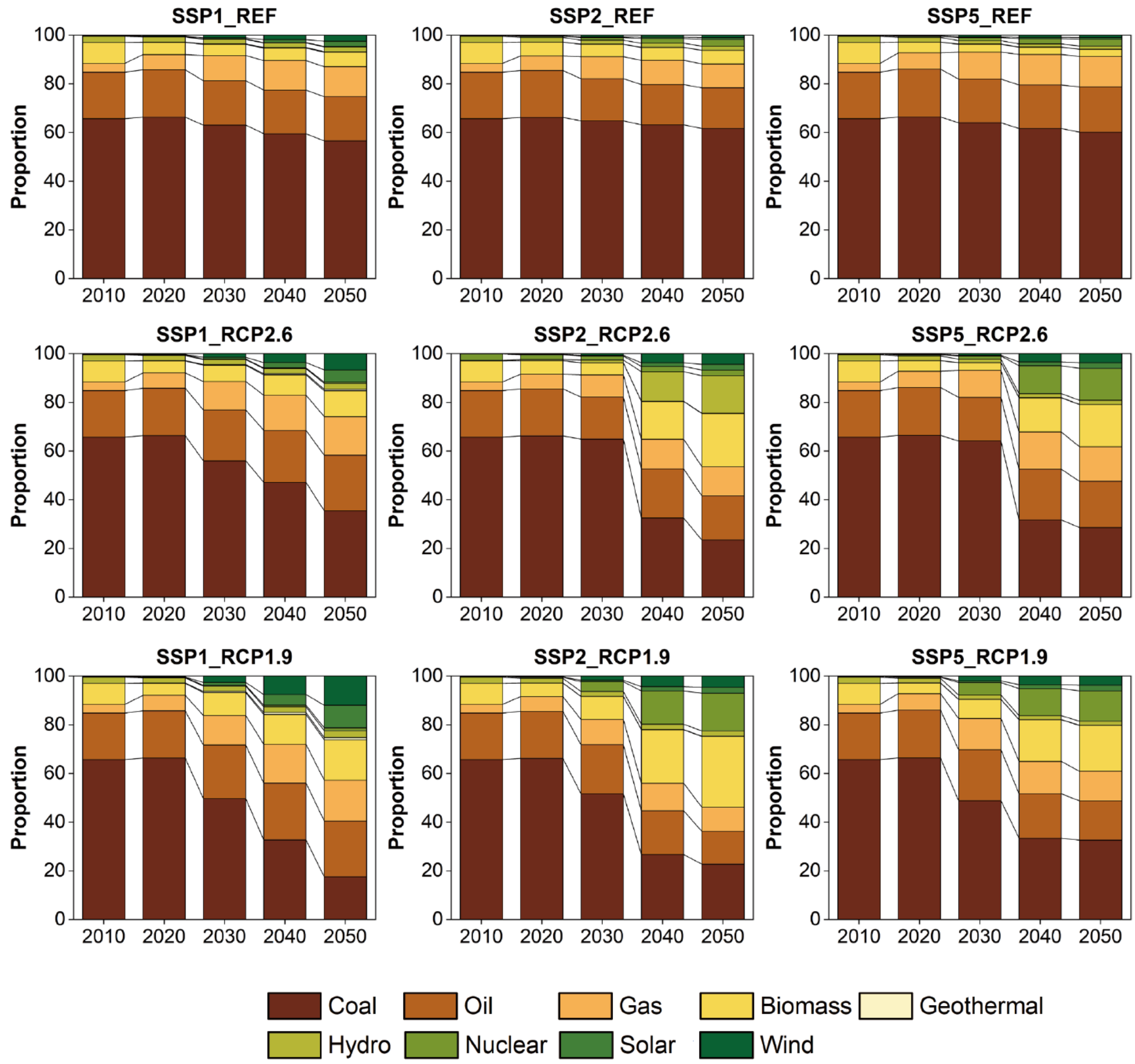

Supplementary Figure 2. Structural changes in China's primary energy consumption under the nine scenarios from 2010 to 2050. Proportions (%) of primary energy in total consumption are shown as stacked columns for each scenario and year.

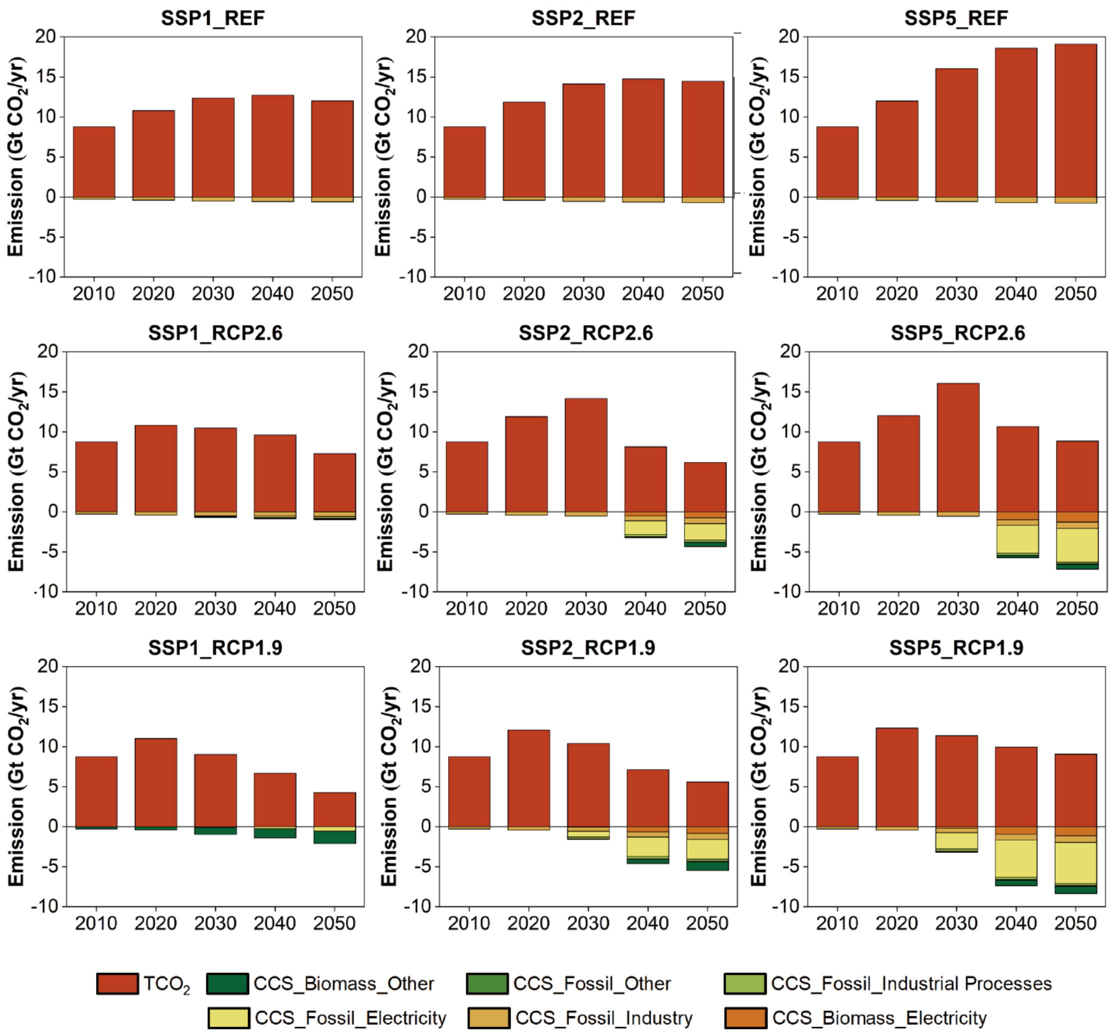

Supplementary Figure 3. Total carbon emission and emission stored by CCS technology under 9 scenarios from 2010 to 2050. TCO_2 represents the total emission, while others represent carbon emissions that absorbed and stored by CCS technology in various sectors.

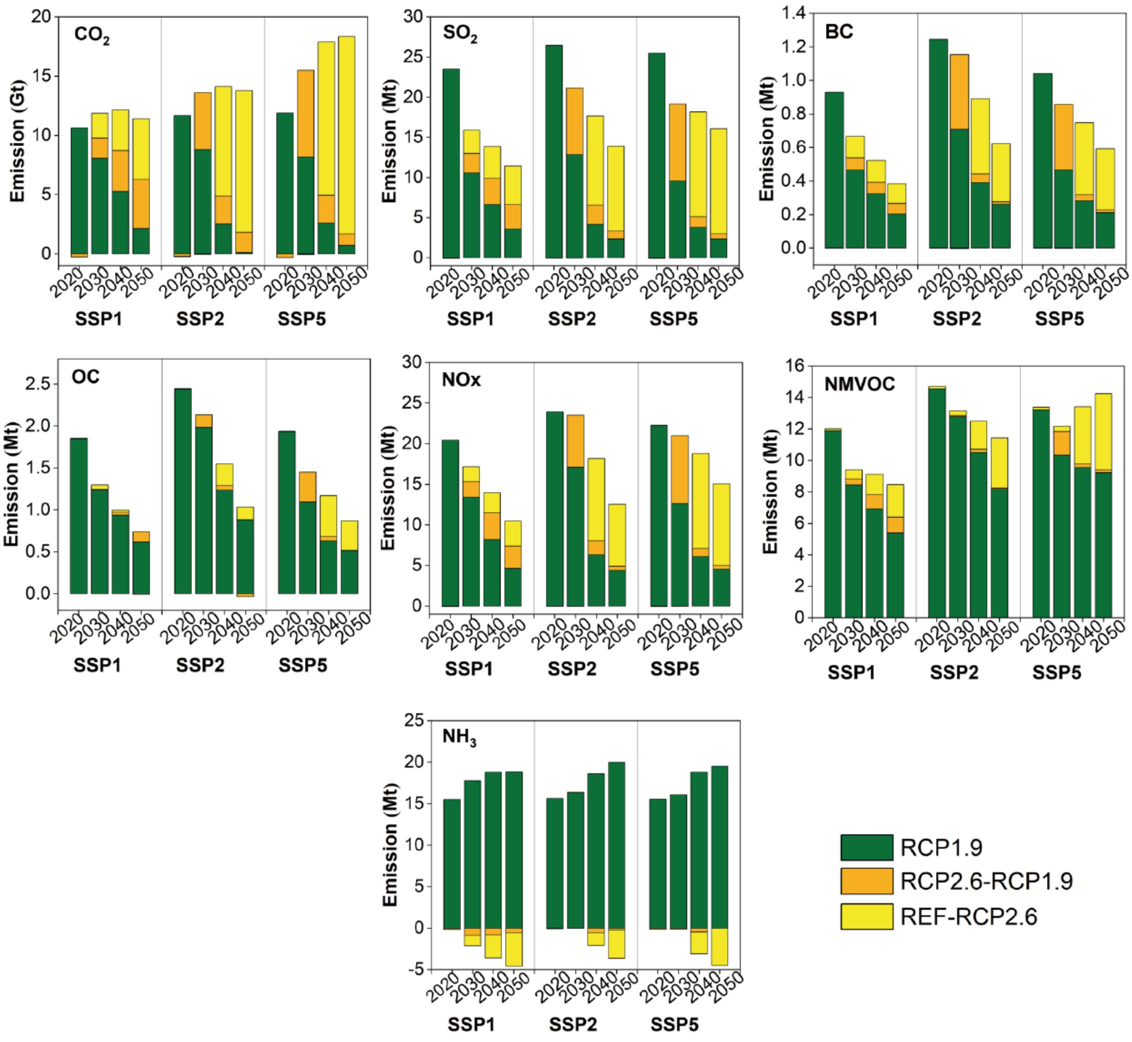

Supplementary Figure 4. CO_2 emission reduction by climate policies and the co-reduction in SO_2, BC, OC, NO_X, NMVOC, NH_3 under various SSP pathways from 2020 to 2050. The green columns represent emission in RCP1.9. The yellow and orange columns represent emission mitigations by switching climate policy from REF to RCP2.6 and from RCP2.6 to RCP1.9, respectively.

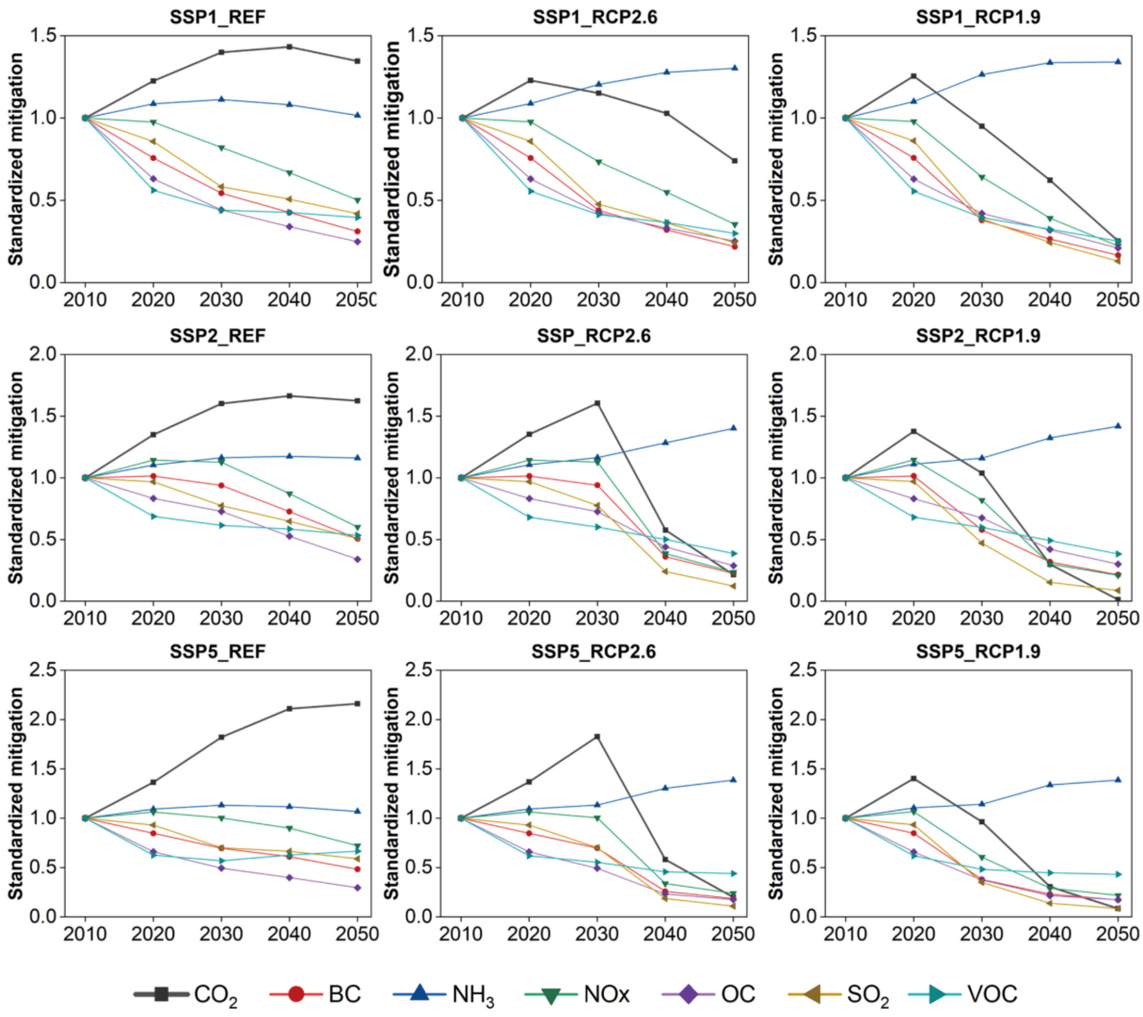

Supplementary Figure 5. Trends of emission changes in CO_2, BC, NH_3, NOx, OC, SO_2, and NMVOC from 2010 to 2050 in China. Emissions are standardized through dividing them by their corresponding emissions in 2010.

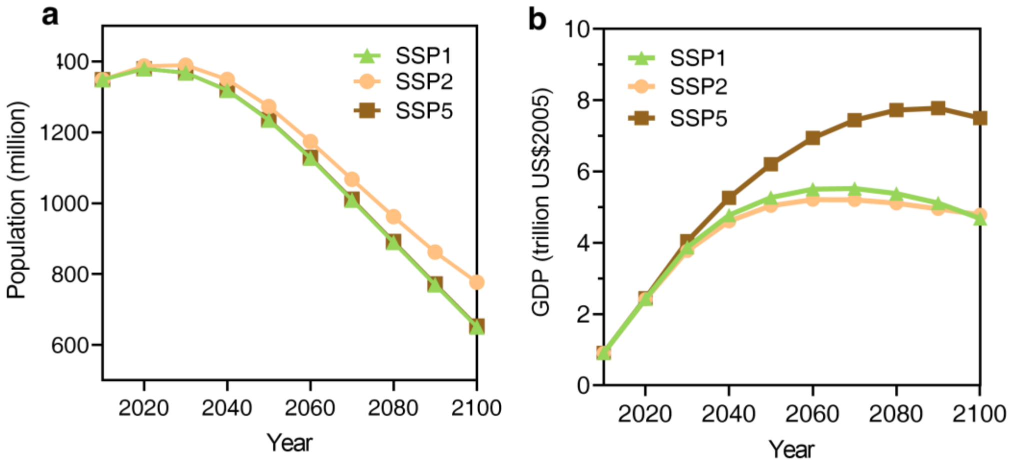

Supplementary Figure 6. Trends in population and GDP under SSPs pathways in China between 2010-2100. a. Population under SSPs pathways in China between 2010-2100 at 10-year intervals; **b.** Same as **a.** but for GDP. The green, yellow and brown markers represent SSP1, SSP2 and SSP5, respectively.

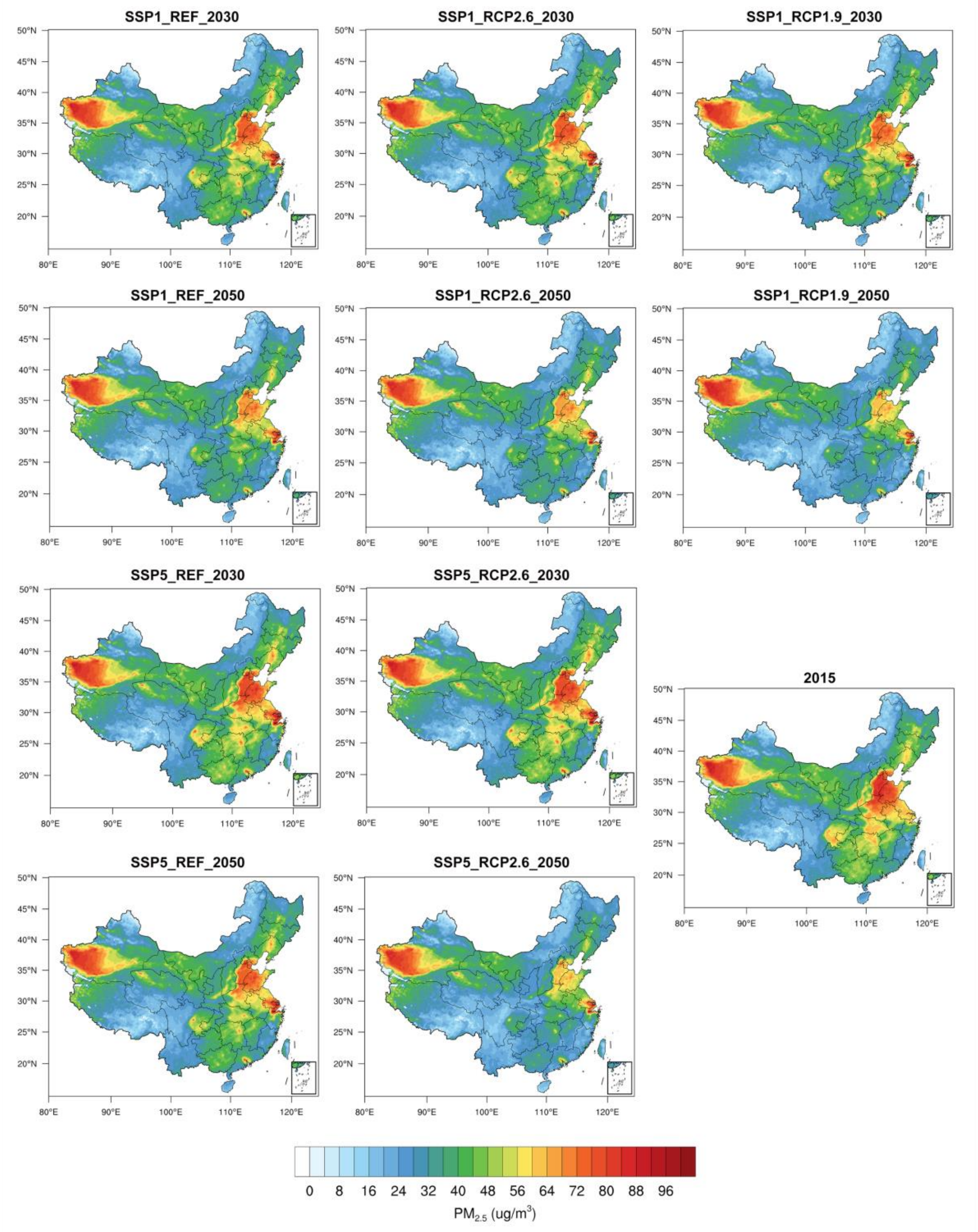

Supplementary Figure 7. Annual mean $PM_{2.5}$ concentration in each scenario and year. Distributions of annual mean $PM_{2.5}$ concentration (unit:μg/m^3) in China are obtained by WRF-Chem simulation and satellite retrival calibration, which are applied for health impact assessment.

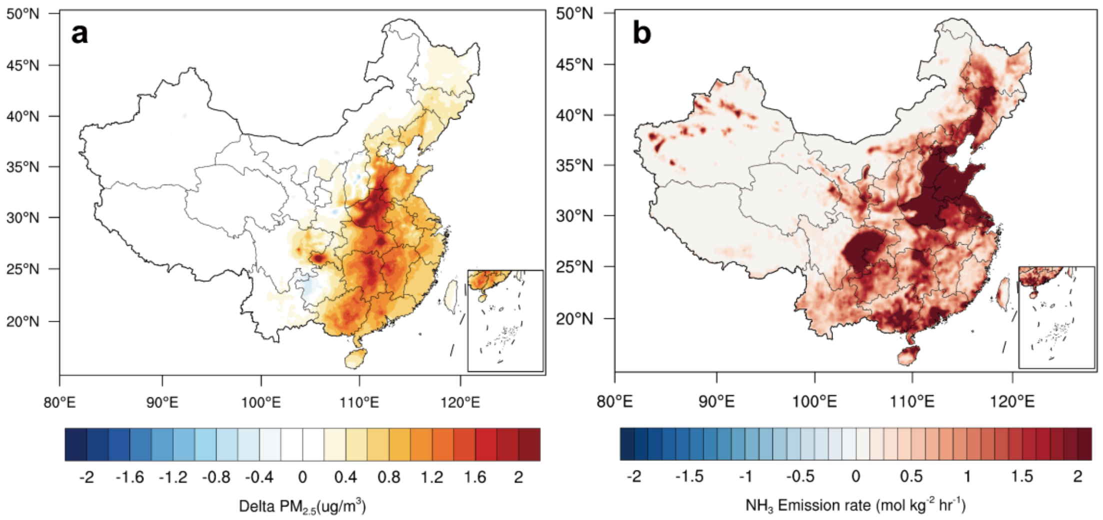

Supplementary Figure 8. Difference in simulated $PM_{2.5}$ concentration and ammonia emission rate between SSP1_REF and SSP1_RCP2.6 in 2030. a. The difference in simulated $PM_{2.5}$ concentration; **b.** The difference in ammonia emission rate. Note that the difference here is SSP1_RCP2.6 minus SSP1_REF.

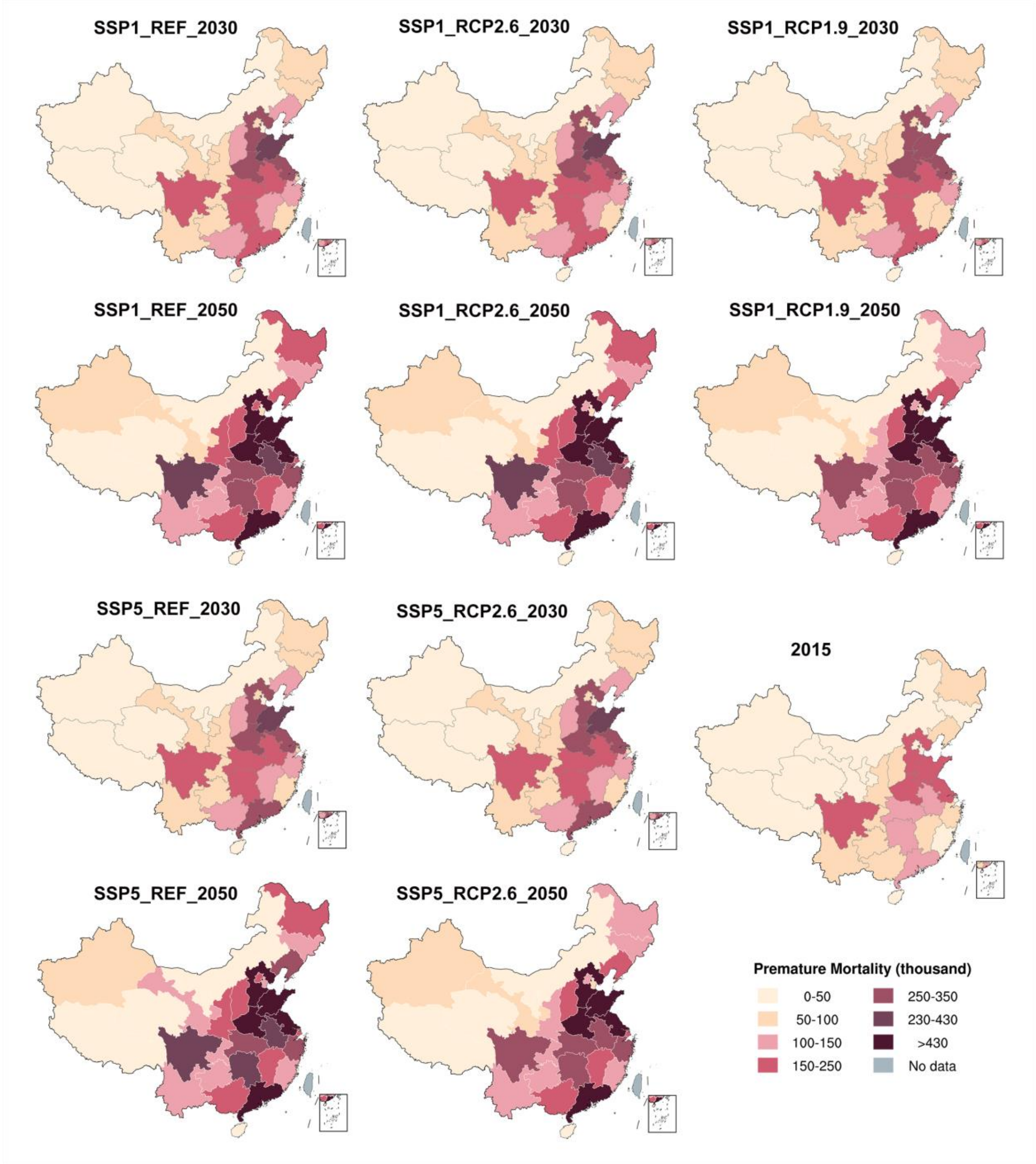

Supplementary Figure 9. Provincial level $PM_{2.5}$-related premature mortality in each scenario and year. The $PM_{2.5}$-related deaths are calculated by GEMM model.

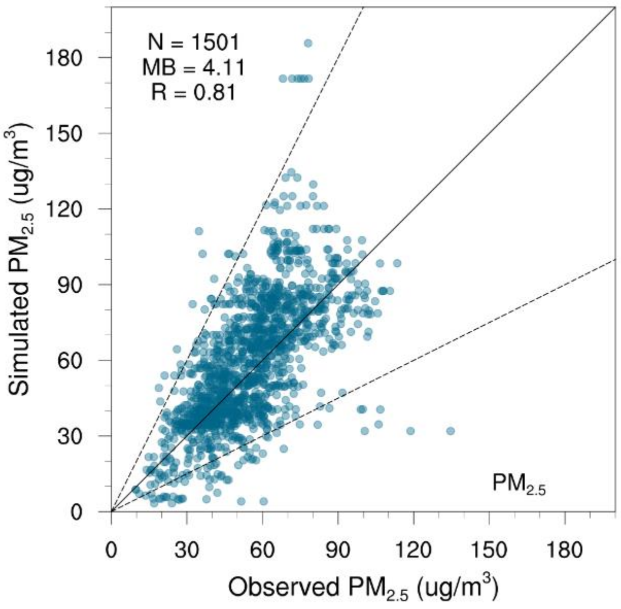

Supplementary Figure 10. Evaluation of simulated annual $PM_{2.5}$ concentrations against ground-based observations. The solid line corresponds to the 1:1 line, and the dashed lines correspond to the 1:2 and 2:1 line. MB and R stand for mean bias and the correlation coefficient, respectively.

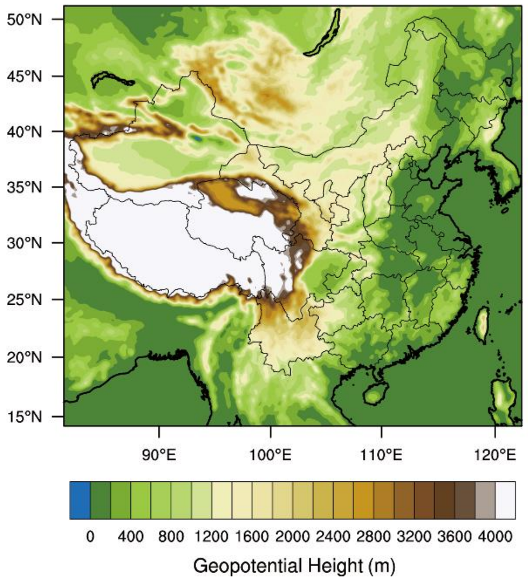

Supplementary Figure 11. Domain setting in WRF-Chem for the parallel experiments. The colour contour shows the spatial distribution of geopotential height.

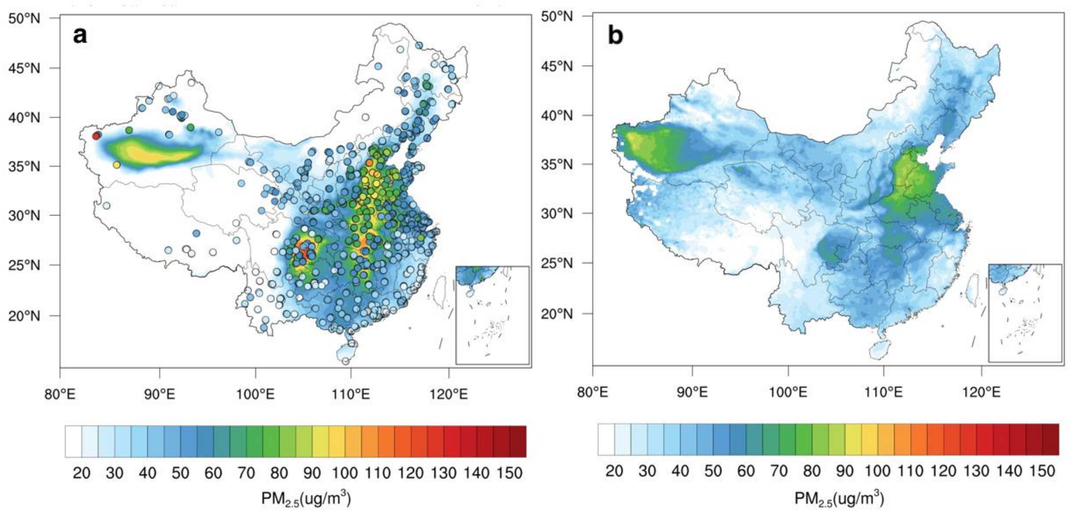

Supplementary Figure 12. Spatial distribution of annual mean $PM_{2.5}$ concentration in 2015. a. Annual mean WRF-Chem $PM_{2.5}$ simulation overlaid with $PM_{2.5}$ observations at air quality monitoring sites; **b.** Satellite retrieved $PM_{2.5}$ concentration by Ma et al.

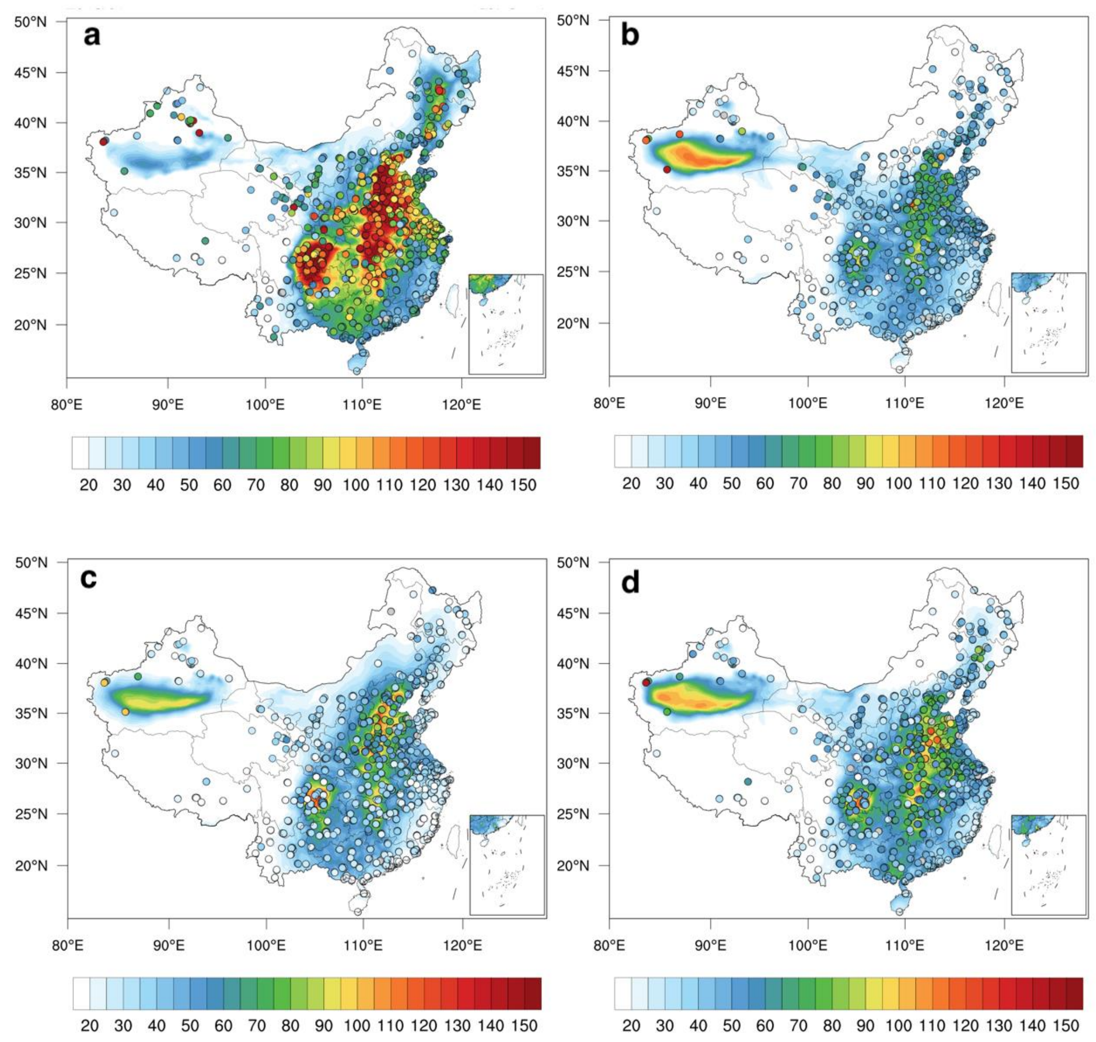

Supplementary Figure 13. Seasonal variation and spatial distributions of simulated $PM_{2.5}$ in 2015. Spatial distributions of monthly mean $PM_{2.5}$ concentration (unit: μg/m^3) in WRF-Chem simulations are overlaid with observations at air quality monitoring sites. **a.** Monthly mean $PM_{2.5}$ in January; **b, c, d** are same as **a**, but for April, July, October, respectively.

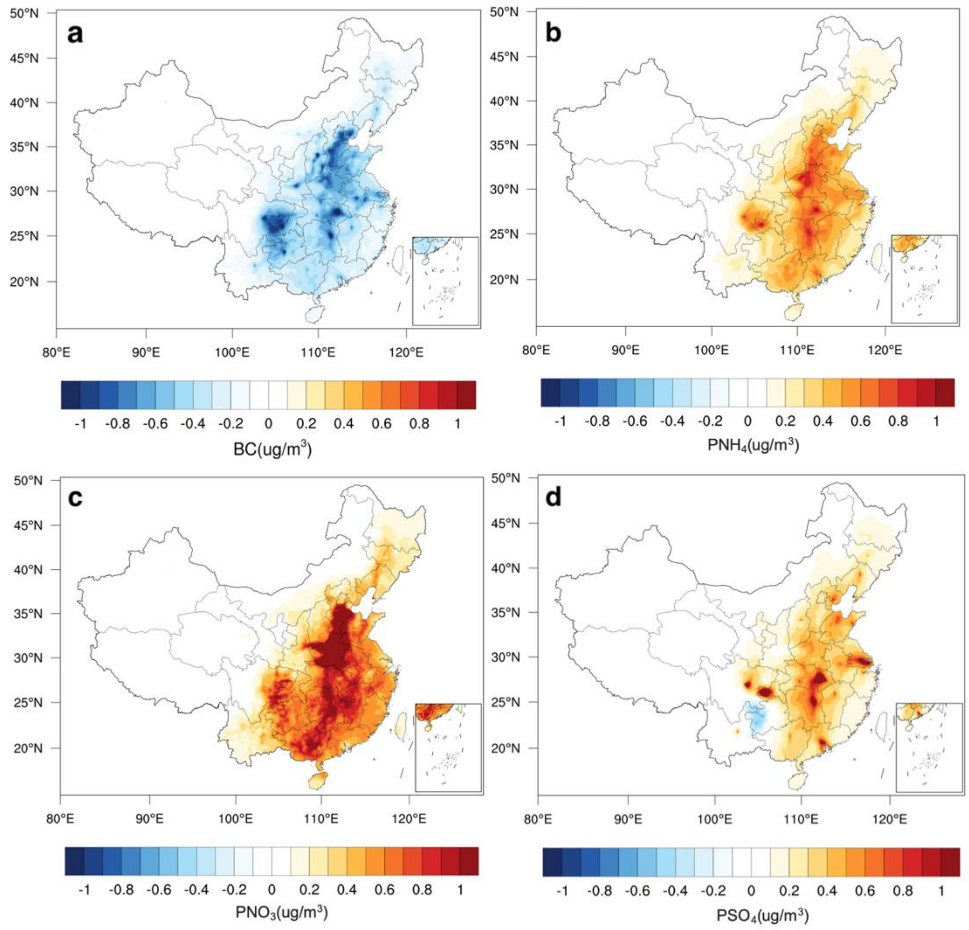

Supplementary Figure 14. Difference in concentrations of $PM_{2.5}$ components in SSP1_REF_2030 and SSP1_RCP2.6_2030. a, BC; **b,** Ammonium; **c,** Nitrate; **d,** Sulfate.

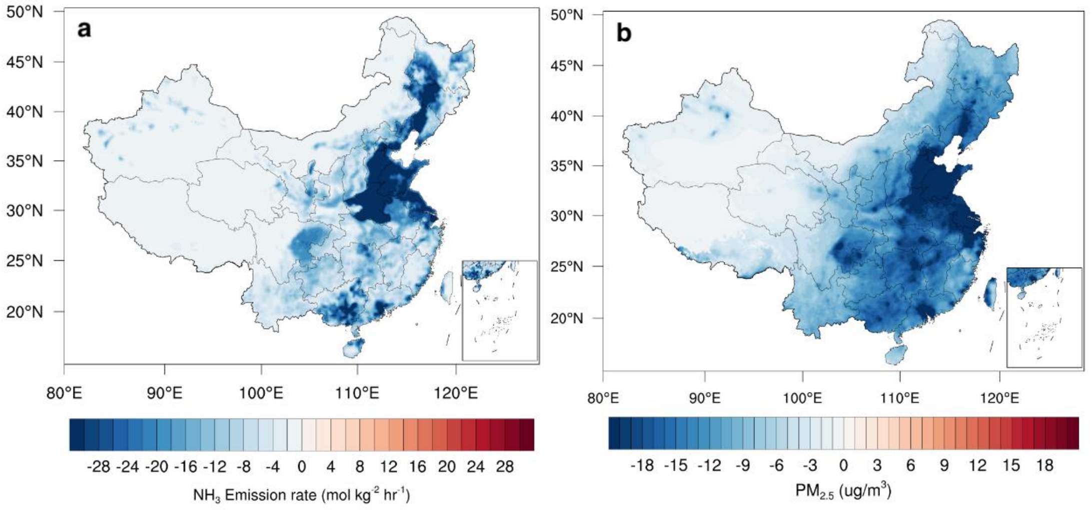

Supplementary Figure 15. Ammonia emission reduction and corresponding decrease in $PM_{2.5}$ concentration. a. Reductions in ammonia emission rate in SSP1_RCP2.6_cutNH_3 relative to SSP1_RCP2.6 in 2030. **b.** Similar to **a.,** but for reductions in $PM_{2.5}$ concentration.

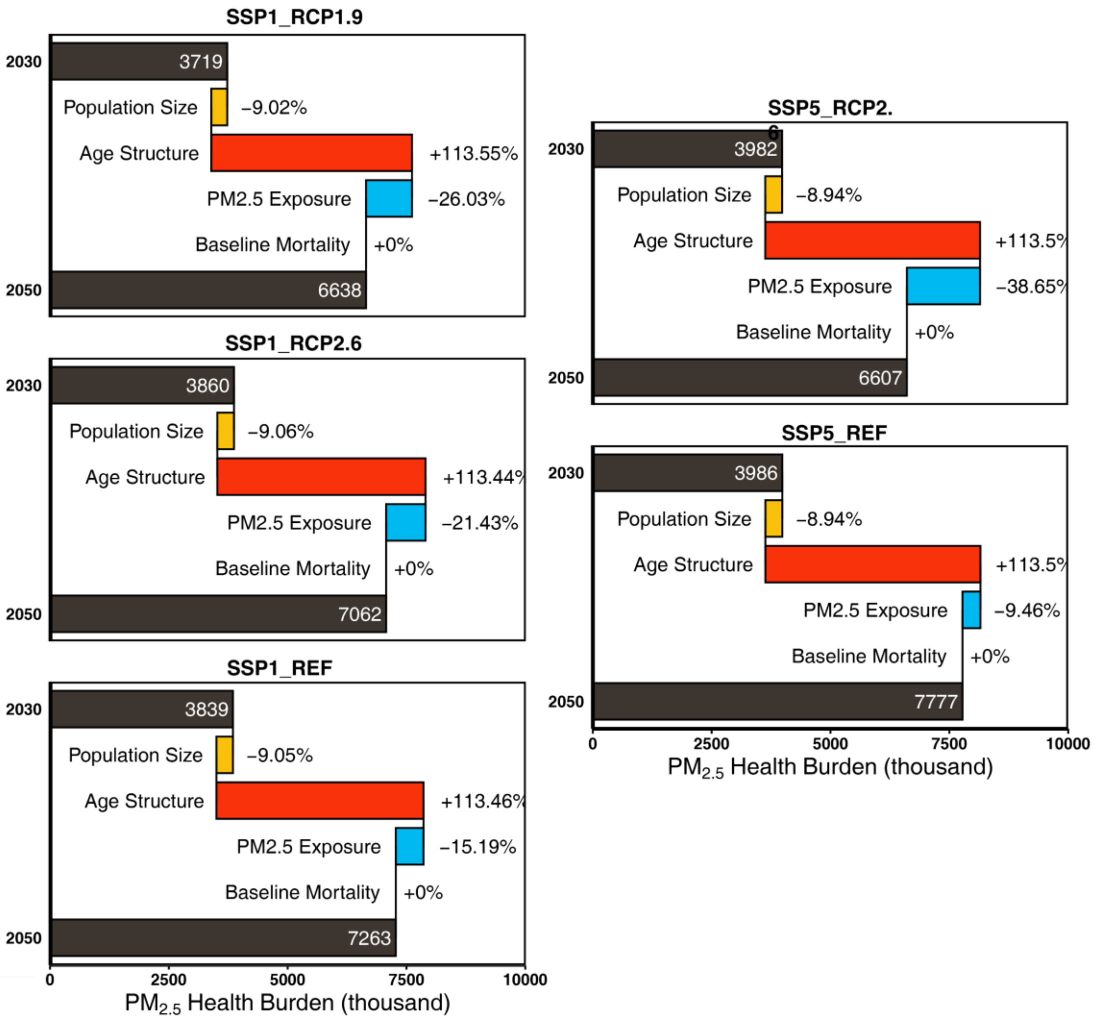

Supplementary Figure 16. Decomposition of the changes in $PM_{2.5}$-related health burden from 2030 to 2050 in SSP-RCP scenarios. Population size, age structure, $PM_{2.5}$ exposure and baseline mortality are the four key factors selected in the decomposition.

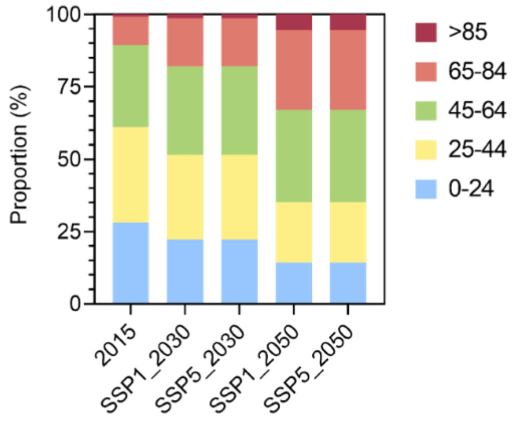

Supplementary Figure 17. Age structure of population in 2015, 2030 and 2050. Proportions (%) of population for each age group are shown as stacked columns for each scenario and year.

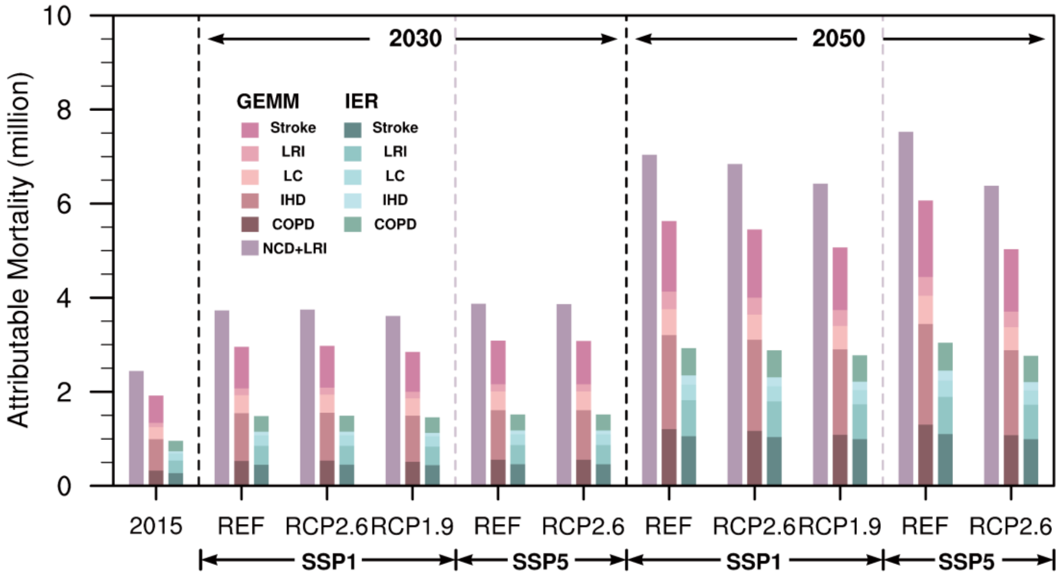

Supplementary Figure 18. $PM_{2.5}$ attributable mortality estimated by GEMM_NCD+LRI, GEMM (with 5 disease terminals) and IER in 2015, 2030 and 2050 under various SSP_RCP scenarios in China. Note that relative risk in GEMM was applied for adults over 25 years old.

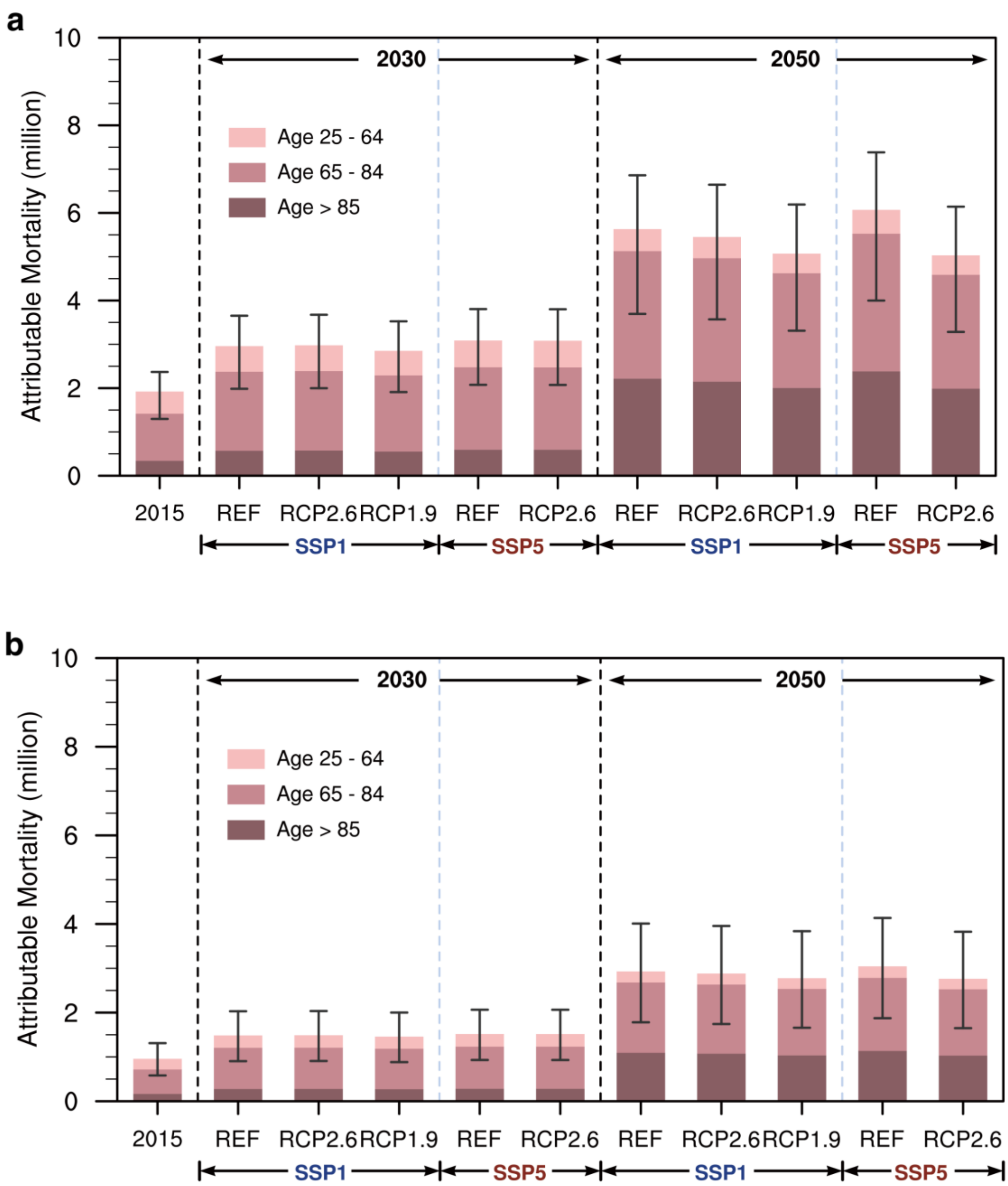

Supplementary Figure 19. Age specific $PM_{2.5}$ attributable mortality estimated by GEMM and IER with 5 disease terminals in 2015, 2030 and 2050 under various SSP_RCP scenarios in China. a. GEMM with 5 disease terminals. **b.** IER. Data are presented as "mean values" with uncertain intervals based on the 95% CIs of RR. The "mean value" is the national sum of gridded deaths calculated based on mean level RR derived from GEMM and IER, respectively. The defined 95% CIs for GEMM and IER model are stated in Supplementary Note 5.1. Note that relative risk in GEMM was applied for adults over 25 years old.

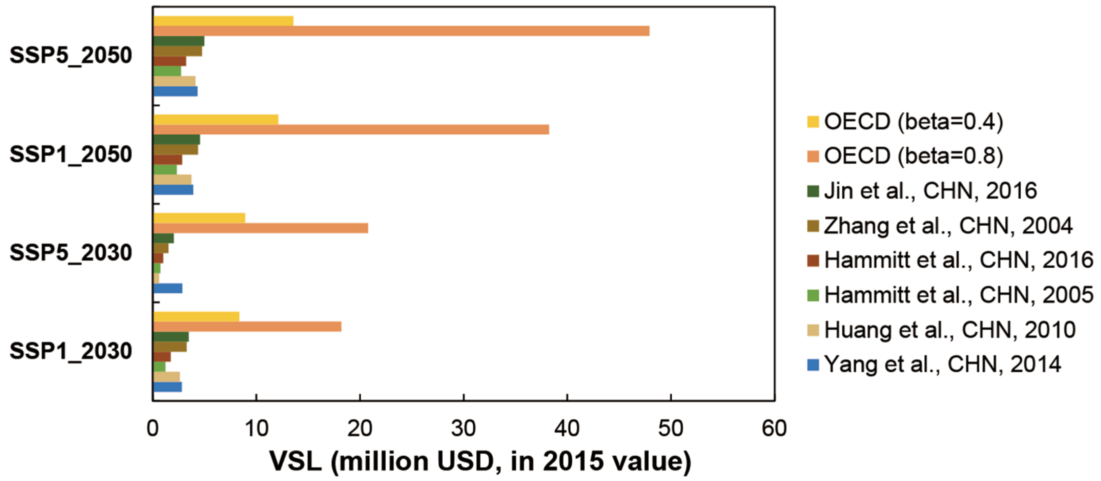

Supplementary Figure 20. Comparison of VSL in China in different SSPs scenarios and years, using various methods and parameters. Different baseline VSLs (OECD: Organization for Economic Co-operation and Development; CHN: China) and income elasticity are applied.

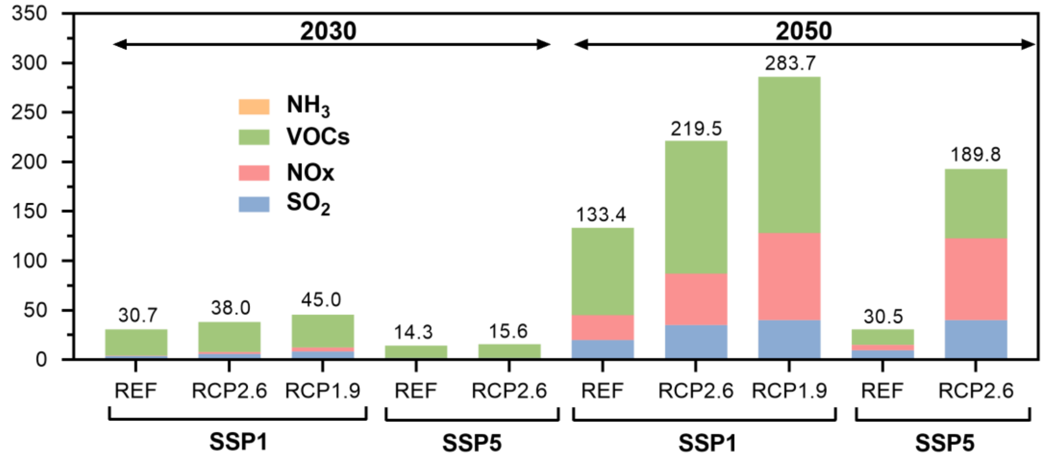

Supplementary Figure 21. Annual avoided abatement cost (unit: billion USD) of air pollution control for all scenarios. Avoided abatement cost for different air pollutants are shown as stacked columns for each scenario and year.

Supplementary Tables

Supplementary Table 1. Peak years of China's carbon emissions and the 2030 carbon emissions intensities under various scenarios.

**Scenarios**	**Peak years**	**Peak emissions** (Mt CO_2/yr)	**Carbon emissions intensity in 2030** (T CO_2/1000USD)
NDC target	2030		0.41~0.47
SSP1_ RCP1.9	~ 2020	10,645	0.21
SSP2 _RCP1.9	~ 2020	11,678	0.26
SSP5 _RCP1.9	~ 2020	11,901	0.19
SSP1_ RCP2.6	~ 2020	10,423	0.25
SSP2 _RCP2.6	~ 2030	13,626	0.4
SSP5 _RCP2.6	~ 2030	15,500	0.36
SSP1_ REF	~ 2040	12,154	0.31
SSP2 _REF	~ 2040	14,124	0.4
SSP5 _REF	~ 2050	18,330	0.36

Supplementary Table 2. Sectoral carbon emissions in China in 2010 Mt CO_2/yr.

Sector			Emission ($Mt\ CO_2/yr$)	Proportion
**Fossil fuels and industry**	Energy demand	Industry	3714.9	43.8%
		Residential and commercial	500.9	5.9%
		Transportation	660.5	7.8%
	Energy supply	Electricity	3399.0	38.9%
		Other	470.9	5.5%
**Land use**			-260.8	-3.1%
**Total**			8485.5	

Supplementary Table 3. Sectors of air pollutant emission sources of China in 2010.

(See excel in figshare: https://figshare.com/articles/dataset/Data_rar/17648891)

Supplementary Table 4. Carbon and air pollutant emissions over time in the SSPs-RCPs scenarios.

(See excel in figshare: https://figshare.com/articles/dataset/Data_rar/17648891)

Supplementary Table 5. Carbon and air pollutant emissions from key sectors over time in the SSPs-RCPs scenarios.

(See excel in figshare: https://figshare.com/articles/dataset/Data_rar/17648891)

Supplementary Table 6. Population-weighted average $PM_{2.5}$ concentration (PWC-$PM_{2.5}$) and national average $PM_{2.5}$ under all scenarios (unit: μg/m^3).

	**PWC-$PM_{2.5}$**	**National mean $PM_{2.5}$**
Base 2015	52.97	38.73
SSP1_REF_2030	53.02	37.67
SSP1_RCP2.6_2030	53.39	37.85
SSP1_RCP1.9_2030	50.92	36.63
SSP5_REF_2030	55.56	38.89
SSP5_RCP2.6_2030	55.49	38.85
SSP1_REF_2050	48.68	35.26
SSP1_RCP2.6_2050	46.98	34.39
SSP1_RCP1.9_2050	43.44	33.04
SSP5_REF_2050	52.94	37.25
SSP5_RCP2.6_2050	43.03	32.50

Supplementary Table 7. GEMM and IER results of premature death (person).

Scenarios	GEMM - NCD&LRI	GEMM - 5ill	IER
**2015**	2,441,900 (2,052,000 ~ 2,809,100)	1,921,000 (1,297,300 ~ 2,369,300)	958,600 (582,300 ~ 1,311,200)
**SSP1_REF_2030**	3,728,600 (3,131,700 ~ 4,291,200)	2,957,400 (1,985,200 ~ 3,653,100)	1,485,500 (904,600 ~ 2,030,800)
**SSP1_RCP2.6_2030**	3,749,200 (3,149,500 ~ 4,314,400)	2,976,100 (1,998,300 ~ 3,675,200)	1,490,100 (908,500 ~ 2,035,700)
**SSP1_RCP1.9_2030**	3,610,100 (3,029,900 ~ 4,157,900)	2,849,900 (1,910,000 ~ 3,525,200)	1,458,100 (882,600 ~ 2,001,000)
**SSP5_REF_2030**	3,869,600 (3,253,100 ~ 4,449,700)	3,085,500 (2,075,200 ~ 3,804,800)	1,517,400 (930,600 ~ 2,064,800)
**SSP5_RCP2.6_2030**	3,865,900 (3,249,800 ~ 4,445,500)	3,082,100 (2,072,700 ~ 3,800,900)	1,516,500 (929,900 ~ 2,064,000)
**SSP1_REF_2050**	7,039,600 (5,894,600 ~ 8,125,400)	5,628,400 (3,694,200 ~ 6,859,400)	2,929,100 (1,781,100 ~ 4,009,400)
**SSP1_RCP2.6_2050**	6,841,400 (5,725,200 ~ 7,901,300)	5,448,400 (3,570,200 ~ 6,644,300)	2,880,100 (1,742,600 ~ 3,955,200)
**SSP1_RCP1.9_2050**	6,426,000 (5,370,600 ~ 7,430,900)	5,070,700 (3,310,800 ~ 6,191,300)	2,774,900 (1,658,600 ~ 3,837,100)
**SSP5_REF_2050**	7,525,500 (6,310,500 ~ 8,674,200)	6,069,400 (3,999,100 ~ 7,383,500)	3,044,400 (1,874,100 ~ 4,136,000)
**SSP5_RCP2.6_2050**	6,380,500 (5,331,800 ~ 7,379,400)	5,030,300 (3,283,000 ~ 6,142,600)	2,763,400 (1,649,300 ~ 3,824,500)

Supplementary Table 8. Premature death (person) in provincial level by GEMM.

(See excel in figshare: https://figshare.com/articles/dataset/Data_rar/17648891)

Supplementary Table 9. Narratives for REF, RCP2.6 and RCP1.9.

	Description
REF	Without explicit climate policies.
RCP2.6	Radiative forcing at ~2.6 W/m^2 by 2100.
RCP1.9	Radiative forcing at ~1.9 W/m^2 by 2100.

Supplementary Table 10. Narratives for all the five SSPs [5].

(See excel in figshare: https://figshare.com/articles/dataset/Data_rar/17648891)

Supplementary Table 11. Energy demand assumptions across SSPs in GCAM.

	SSP1	SSP2	SSP3	SSP4	SSP5
Fuel Preference Elasticity for Traditional Bioenergy	-2.5	-2	-1	-0.75	-2.5

Supplementary Table 12. Technical change on extraction cost (% per year) of fossil fuel across SSPs in GCAM.

Fuel	SSP1	SSP2	SSP3	SSP4	SSP5
Coal	0.5%	0.5%	1%	0.5%	2%
Gas	0.5%	0.5%	0.5%	1%	2%
Conventional oil	0.5%	0.5%	0.5%	1%	2%
Unconventional oil	0%	0.5%	0.5%	2%	

Supplementary Table 13. Food demand in SSP-RCP scenarios in China [9].

Scenarios	2030					2050				
	SSP1			SSP5		SSP1			SSP5	
	REF	RCP2.6	RCP1.9	REF	RCP2.6	REF	RCP2.6	RCP1.9	REF	RCP2.6
Per capita food demand	3360	3334	3285	3345	3317	3476	3363	3255	3300	3224
Livestock	756	751	744	731	727	766	746	725	695	678
Crops	2604	2583	2542	2614	2590	2711	2618	2530	2605	2546

Unit: kcal/cap/day

Supplementary Table 14. Agricultural production and land use in future China [9].

Scenarios	2030					2050				
	SSP1			SSP5		SSP1			SSP5	
	REF	RCP2.6	RCP1.9	REF	RCP2.6	REF	RCP2.6	RCP1.9	REF	RCP2.6
**Agricultural Production**	958	1159	1360	990	1011	1087	1594	1766	1117	2371
Crops	659	761	846	686	691	660	1007	1104	698	1602
**Crops | Energy**	49	61	82	48	51	103	162	210	83	654
Crops | non-energy	609	701	764	638	640	557	845	894	616	948
Residues | Energy	181	280	396	181	196	277	437	512	252	603
Waste | Energy	86	87	87	92	92	122	122	122	138	139
**Land Cover**	938	938	938	938	938	938	938	938	938	938
Cropland	105	117	140	110	111	89	124	145	98	175
**Energy crops**	3	4	6	3	4	7	11	15	5	44
Forest	191	197	195	186	186	202	228	229	196	204
Pasture	394	381	366	396	396	397	353	337	397	335

Unit: million t DM/yr for agricultural production; **million ha** for land use.

Supplementary Table 15. Annual total emissions (unit: Mt) of CO_2 and air pollutants in scenario SSP1_REF_2030 and SSP1_RCP2.6_2030 from GCAM.

Species	SSP1_REF_2030	SSP1_RCP2.6_2030
CO_2	11873.20	9772.06
BC	0.67	0.54
CO	43.039	40.98
NH_3	15.66	16.94
NO_X	17.13	15.33
OC	1.30	1.24
SO_2	15.90	13.00
VOC	9.41	8.83

Supplementary Table 16. Design of experiments for sensitivity test of ammonia emission control.

Scenario	Year	Emission setting
SSP1_RCP2.6_cutNH_3	2030	50% ammonia cut from agriculture sector
SSP1_RCP1.9_cutNH_3	2030	

Supplementary Table 17. National annual mean $PM_{2.5}$ in scenarios with or without additional NH_3 control.

Scenario	Year	$PM_{2.5}$ concentration (μg/m^3)	Population-weighted $PM_{2.5}$ concentration (μg/m^3)
SSP1_REF	2030	37.67	53.02
SSP1_RCP2.6	2030	37.85	53.39
SSP1_RCP1.9	2030	36.63	50.92
SSP1_RCP2.6_cutNH_3	2030	30.09	34.73
SSP1_RCP1.9_cutNH_3	2030	29.71	33.89

Supplementary Table 18. Comparison of costs and benefits of climate policies using health impact results by IER model.

		Health benefit (billion USD)			Cost (billion USD)
		Local VSL*	Scaled international VSL*		
			β = 0.8	β = 0.4	
RCP2.6_2030	SSP1	-8.08 (-16.21~-5.73)	-66.39	-30.49	27.21
	SSP5	0.84 (0.51~2.40)	13.63	5.86	0
RCP2.6_2050	SSP1	138.79 (114.23~224.26)	1463.95	463.93	167.91
	SSP5	908.36 (767.55~1398.48)	10523.82	2978.70	684.03
RCP1.9_2030	SSP1	47.45 (33.71~95.26)	389.92	179.09	—
RCP1.9_2050	SSP1	436.50 (359.27~705.30)	4604.14	1459.08	—

Supplementary Table 19. VSL values from existing local VSL studies in China.

	VSL (million $)	INC (million $)	City	Year	MVSL
Yang et al.[62]	1.92	0.00511	Nanjing	2014	99.794 [61]
Huang et al.[63]	1.60	0.00374	Beijing	2010	
Hammitt et al.[64]	0.06	0.00225	Chengdu	2005	
Hammitt et al.[64]	0.93	0.00601	Chengdu	2016	
Zhang et al.[60]	2.07	0.00209	Chongqing	2004	
Jin et al.[65]	2.68	0.00601	Beijing	2016	

*Note that: INC represents the per capita disposable income, which sources from China Statistical Yearbooks. Both the VSL and INC in baseline years (shown above) are in 2017 $ price, i.e. adjusted by 2017 PPP (Purchasing Power Parity).

Supplementary Table 20. Air pollutant reduction ratios from the 2010 level.

Year	Scenarios	SO_2	NO_X	NMVOCs	NH_3
2030	SSP1_REF	-0.4172	-0.1792	-0.5602	0.1123
	SSP1_RCP2.6	-0.5236	-0.2657	-0.5873	0.2037
	SSP1_RCP1.9	-0.6137	-0.3581	-0.6051	0.2642
	SSP5_REF	-0.2998	0.0037	-0.4315	0.1320
	SSP5_RCP2.6	-0.2982	0.0051	-0.4469	0.1336
2050	SSP1_REF	-0.5809	-0.4980	-0.6043	0.0152
	SSP1_RCP2.6	-0.7573	-0.6459	-0.7010	0.3022
	SSP1_RCP1.9	-0.8689	-0.7773	-0.7476	0.3409
	SSP5_REF	-0.4110	-0.2788	-0.3335	0.0690
	SSP5_RCP2.6	-0.8904	-0.7600	-0.5602	0.3862

Supplementary Table 21. Cost savings by climate policies in air pollution control.

Year	SSPs	Climate policy	Cost saving (Billion USD)
2030	SSP1	RCP2.6	7.3
		RCP1.9	14.4
	SSP5	RCP2.6	1.3
2050	SSP1	RCP2.6	86.2
		RCP1.9	150.3
	SSP5	RCP2.6	159.2

Supplementary Note 1: Mitigation pathways under SSP-RCP scenarios

1.1 Representative Concentration Pathways (RCPs) and Shared Socioeconomic Pathways (SSPs)

Scenarios about future socioeconomic and climate developments are used to study the scope and implications of climate change and responses to it [1].The Representative Concentration Pathways (RCPs) covers the climate forcing dimension of different possible futures, allowing subsequent analysis by both Climate models and Integrated Assessment Models (IAMs) [2]. The RCPs reach different levels of radiative forcing in the year 2100 and thus can serve as proxy for climate targets. Hence, IAMs could explore a range of different technological, socio-economic and policy futures that could lead to a particular concentration pathway and magnitude of climate change. Narratives for the RCPs adopted in this study are listed in Supplementary Table 9.

To characterize socioeconomic challenges to mitigation and adaptation in a reference case without explicit climate policies and without consideration of climate change impacts[1], the SSPs are introduced in O'Neill et al. (2014). The SSPs provide five distinctly different pathways about future social, economic and environmental developments, spanning a range of challenges to greenhouse gas (GHG) emissions mitigation and adaptation to climate change [4]. They are defined as reference pathways describing plausible alternative trends in the evolution of society and ecosystems over a century timescale, in the absence of climate change or climate policies [3]. The SSPs vary in assumptions on energy and land use changes, and also assumptions on regional pollution, in particular air and water. Narrative assumptions for the SSPs are listed in Supplementary Table 10, in which the pathways adopted in this article are highlighted in color.

1.2 SSP-RCP framework and attainability of scenarios

In fact, to be useful for climate policy analysis, the scenarios should include both climate policies and others that are not directly related to climate. All those policies controlled by non-climate objectives will either have a substantial impact on climate policy related outcomes or be substantially impacted by climate policy itself [6]. The scenario matrix SSP-RCP is defined by combinations of distinct SSPs and climate forcing outcomes (as characterized by the RCPs).

The SSP-RCP framework facilitates the coupling of multiple socioeconomic reference pathways with climate model products using the representative concentration pathways, allowing for improved assessment of climate impacts, adaptation and mitigation [6].

The RCPs and SSPs are paired by imposing a set of climate policies, called Shared Climate Policy Assumptions (SPAs), on the SSP baseline. Each SSP has its own SPA consistent with the narrative from which that SSP was developed. These SPAs describe the policy environment in both the near and long term. The long-term mitigation target as determined by the long-term forcing in an RCP will be a central part of the quantitative information given in the SPAs. In principle, the target could be specified in a number of ways, ranging from a global temperature target, to a climate forcing target to a cumulative emissions budget for the entire world. It may also include some constraints on the pathway of climate forcing or global emissions [7].

The set of climate policy assumptions will have strong implications for the outcome of the scenario analysis, including energy structure, carbon trajectories, etc. Then, by comparing scenarios with and without climate policies, the impact of climate policy can be isolated from the other factors that are changing simultaneously (e.g., population, income, income distribution, land-use).

Studies have explored the feasibility of limiting the end-of-century radiative forcing to 2.6 W/m^2 or 1.9 W/m^2 under the five SSPs, using six integrated assessment models (IAMs) [8]. Some, but not all, SSPs are amenable to pathways to 2°C and 1.5°C.

Successful 1.9 W/m^2 scenarios are characterized by a rapid shift away from traditional fossil-fuel use towards large-scale low-carbon energy supplies, reduced energy use, and carbon-dioxide removal.

The SSP1 assumptions include sustainable consumption patterns, low population growth, energy efficiency improving faster than historically, rapid deployment of renewable energy, and global cooperation. With rapid technology diffusion and effective global climate policy from 2020 onwards, all participating models were able to create scenarios in line with an end-of-century forcing target of 1.9 W/m^2. As for SSP5, the ability to successfully deploy negative emissions technologies and the potential to replace technologies with significant amounts of residual CO_2 emissions appear a key determining factor in making it possible for models to counterbalance the otherwise high energy and resource intensity assumed by the SSP5 narrative. Under these assumptions, the SSP5-RCP1.9 scenario has been successfully simulated in GCAM model [9] and REMIND [7].

1.3 CO_2 mitigation pathways

Carbon mitigation can be achieved through a wide portfolio of measures in the energy, industry and land-use sectors, which are the main sources of carbon emissions in China (Supplementary Table 2).

Energy structure adjustment is an important way to reduce carbon emissions. The structural changes include replacement of carbon-intensive fossil fuels by cleaner and renewable energy on the supply side, while refer to energy conservation, efficiency improvements and also the electrification on the demand side. According to Supplementary Fig. 2, changes in primary energy consumption between SSPs baseline scenarios, i.e. SSP_REF, are far less than that brought by the implementation of climate policies. Under both the REF and RCP2.6 scenarios, the proportion of fossil fuels in primary energy consumption would keep above 80% and only decrease slightly after reaching the peak around 2020 or 2030. Though the share of coal and oil will decline

slightly, increases in natural gas use would play the major role in the growth of fossil fuels consumption before 2030. Under the RCP2.6 scenarios, the proportion of oil and natural gas will remain stable at ~20% and ~13%, respectively, after 2030, and the proportion of coal will decline rapidly, from 56~65% in 2030 to 23~35% in 2050. Under the RCP1.9 scenarios, China could possibly fulfill the NDC commitment of improving the share of non-fossil fuels to ~20% in the total primary energy consumption in 2030, with more ambitious climate target.

Meanwhile, the preference of cleaner alternatives varies between the SSP scenarios under the climate targets. For the SSP1 scenarios, coal is largely replaced by clean and renewable energy, i.e., solar and wind energy, especially under the 1.5°C target. Yet, the SSP2 and SSP5 scenarios mainly rely on biomass and nuclear energy as non-fossil energy supply to achieve ambitious goals.

Aside from energy structural changes, application of CCS technologies is another critical way to achieve temperature control objectives (Supplementary Fig. 3). For all scenarios, CCS technology is to some extent a determinant to achieve the mitigation goals of carbon emissions, especially for those rigorous climate targets. Under SSP1_RCP2.6 scenario, the CCS technology would be applied to store 719 Mt CO_2 /yr ~1003 Mt CO_2 /yr from 2030 to 2050. Under the SSP2_RCP2.6 and SSP5_RCP2.6 scenarios, CCS technologies are assumed to be widely used after 2030, which would respectively store 40% and 53% of the total CO_2 emissions in 2040, and 70% and 81% in 2050. Under the SSP1_RCP1.9 scenario, nearly half of the total CO_2 emissions by 2050 will be captured and stored, with CCS applied broadly in fossil energy use in industry and power generation. For SSP2 and SSP5 scenarios, almost all (98% and 92%) CO_2 emissions from fossil and biomass fuels use in power generation, industry and other sources will be collected and stored by CCS technology by 2050.

1.4 Mitigation of non-CO_2 GHG emissions

The non-CO_2 GHGs in GCAM include methane (CH_4), nitrous oxide (N_2O) and

fluorinated gases (CFCs, HFCs, SF_6, PFCs), which are initialized from the CEDS inventory[10]. In general, the mitigations of non-CO_2 GHGs are addressed by using parameterized functions for Marginal Abatement Cost (MAC) curves to change emission factors over time in the climate policy scenarios. Here, the MACs are an exogenous input, and are read in as the percent of emissions abated as a function of emissions prices, which are assigned to a wide variety of technologies, mapped directly from US-EPA[1]. Emissions ***E*** are modelled for any given technology in time period **t** as:

$$E_t = A_t \times F_{t0} \times (1 - MAC(Cprice_t))$$

Where, A is activity level (e.g., output of a technology), F_{to} is the emissions factor for base-year emissions per unit activity, *Cprice* is the carbon emission price (i.e., the price of carbon emission rights).

1.5 Air pollutant emissions in SSP-RCP framework from GCAM

The projections of carbon and air pollutant emission in this study are obtained through top-down simulations by GCAM, an integrated assessment model. The impacts of socio-economic development and climate policies are depicted in the model settings, but not specific polices and measures.

Based on these settings from SSPs and RCPs, the model searches for the balance solution of prices that cause all markets to be cleared and all consistency conditions to be satisfied. Thus, the emission curves are determined by both pollutant emission factors set in the SSPs, and the energy or final consumption (i.e., the solution) determined by the SSP-RCP. The air pollutant emissions from key sectors over time from GCAM can refer to Supplementary Table 4 and 5.

(i) Pollutant emission factors set in SSPs

Each SSP represents a different narrative on how the environmental issues are addressed in the future. Then, the emission factors are implemented in the baseline scenarios (i.e. SSPs-REF) that describe the SSP narratives.

The emissions factors for air pollutants in the SSPs are given by quantitative guidelines based on a dataset of regional emission factors for energy-related combustion and transformation sectors until 2030 based on current policies and technological options derived from the GAINS model [12,13]. Meanwhile, the future air pollution controls in the SSPs are mapped into different levels, i.e. strong, medium and weak, considering economical capacity and technological levels. Thus, emissions factors in the future are determined by the baseline dataset and the air pollution control assumption, which is depicted by pollution control targets, economic development and pollution control technologies.

Until 2030, emission factors assumed in the SSPs reflect assumptions on the attitudes to health and environment and the institutional capacity to implement pollution control in the near-term. The dataset of emissions factors until 2030 derived from GAINS [12,13] as mentioned above will be adopted in the medium scenario, while multiplied and scaled values in the weak and strong pollution control scenarios. After 2030, the trajectories depend on the expected status of pollution control (i.e., the extent of technological change and the progress towards "maximum technically feasible reduction" levels of emission factors) and further economic development. More detailed information is described in Rao et al [14].

(ii) RCPs and SSPs jointly determine the final solution

The final energy consumption (i.e. the solution in GCAM) under the SSP-RCP framework is jointly determined by the environmental policy settings in SSPs baselines and the radiation forcing level defined by RCPs.

The long-term radiation forcing target in an RCP will be imposed on the SSPs baselines as quantitative constraints, which is the most significant difference between RCP1.9, RCP2.6 and REF. This set of climate policy assumption will have strong implications for the outcome (i.e. solution) of the scenario analysis, including the carbon trajectories, the energy structure, etc. Besides, the RCP scenarios focus on climate mitigation and do not include extra policies on air pollution control compared

to the baseline scenarios (i.e., SSP-REF)[14].

Apart from emission factors, the SSPs narratives also included some other environmental policies considerations. Since model searches for the solution of energy structure by balanced markets as mentioned above, the phase-out and replacement are done by adjusting related economic parameters. In particular, the schedule for the phase-out of traditional solid fuels can be adjusted by different fuel preference elasticity across SSPs (Supplementary Table 11), the parameter that links fuel preferences to per capita income. More negative fuel preference elasticities result in faster phase outs. Similarly, the replacement of coal in power plants can be determined by varying extraction cost and other cost adders, which can be adjusted by cost-related technology development across SSPs (Supplementary Table 12). Thus, the phase-out of solid fuels and coal-fired power plants are fully considered from the perspective of carbon mitigation, rather than the control target of air pollution.

As above, the difference of pollutant emissions between SSPx-REF and SSPx-RCPs reflects the synergistic effect of climate policies (such as boosting of clean energy, land use change, etc.) on air pollution control.

Supplementary Note 2: Simulations of ambient $PM_{2.5}$ in China

2.1 WRF-Chem model and configuration

WRF-Chem model is a state-of-the-art meteorology/chemistry model with a variety of coupled physical and chemical processes, including emission and deposition, advection and diffusion, gaseous and aqueous chemical transformation, aerosol chemistry and dynamics, etc[15]. In this study, coupled dynamical and chemical simulations were conducted using WRF-Chem, version 3.6.1. Note that we used a modified core program of WRF-Chem based on our previous studies[16], which has incorporated additional pathways of secondary pollution enhancement in China. Domain at a grid resolution of 20km × 20km covers the Greater China region, including mainland China and part of East Asia (Supplementary Fig. 11).

Eleven parallel experiments were designed, including the base run in 2015 and 10 runs for 5 selected scenarios (i.e., SSP1_REF, SSP1_RCP2.6, SSP1_RCP1.9, SSP5_REF and SSP5_RCP2.6), in 2030 and 2050, respectively. For each experiment, the average of daily $PM_{2.5}$ concentrations in 4 representing months (Jan, Apr, Jul and Oct) were regarded as the estimation of annual average $PM_{2.5}$ concentrations. The simulations were split into independent 48-hour runs for meteorological fields, while the chemical outputs from the preceding run were used as the initial conditions for the following run. The first 7 days were regarded as the model spin-up period for atmospheric chemistry to allow the model to reach a state of statistical equilibrium under the applied forcing[17,18]. The initial and lateral boundary meteorological conditions were National Centers for Environmental Prediction (NCEP) global final analysis data (FNL) with a 1°×1° spatial resolution, updated every 6 hours.

Key parameterization options for model were the RRTMG radiation scheme[19] to depict radiation transmission processes, the Noah land surface scheme to describe the land-atmosphere interactions[20], the Yonsei University (YSU) boundary layer scheme[21]

for the main processes of planet boundary layer, and the Lin microphysics scheme[22] together with the Grell-Freitas cumulus parameterization to reproduce the cloud and precipitation processes[23]. For numerical representation of atmospheric chemistry, Carbon-Bond photochemical mechanism was applied, combined with Model for Simulating Aerosol Interactions and Chemistry (MOSAIC) aerosol module.

MIX, the mosaic Asian anthropogenic emission inventory developed by Tsinghua University[24] were applied for the 2015 base run, which has already integrated the Multi-resolution Emission Inventory for China (MEIC). Then, annual total emissions $E_{i,j,s,y}^{GCAM}$ obtained from GCAM model were distributed to grids, assuming that emissions in scenarios exhibit the same spatial and seasonal distribution as that specified in the MIX Asian emission inventory, as Eq. (1):

$$E_{i,j,s,y,g,m} = E_{i,j,s,y}^{GCAM} \times \frac{E_{i,j,g,m}^{MIX2015}}{E_{total}^{MIX2015}}$$

where $E_{i,j,s,y,g,m}$ represent the emission of specie i from sector j, in grid g, month m, scenario s and year y, respectively.

For non-anthropogenic emissions, the estimated biogenic inventory provided by MEGAN (the Model of Emissions of Gases and Aerosols from Nature) was also used [25]. We also used the background field and default dust options in WRF-Chem.

2.2 Modeling the effect of climate policies on $PM_{2.5}$ exposure

In the ideal case, exposure can be estimated using a perturbation in emissions with compared to a reference case to evaluate the impacts of emission change on human exposure [26]. For example, a great number of researches explored the air quality changes and related health impacts caused lockdown measurements during the COVID-19 pandemic, a unique natural experiment [27,28].

Aside from measured concentrations, air quality modeling studies like WRF-Chem were widely applied among them, to analyze the impact of policies or measures

on air pollution [27,29]. We also employ this sensitivity analysis to evaluate the impact of climate policies on the ambient $PM_{2.5}$ and related attributable deaths in China. The spatial change ratios of simulated $PM_{2.5}$ of all scenarios compared to the 2015 base run represent the total impacts of climate policies with different ambitions under various soci-economic paths (SSPs). These spatial change ratios were then multiplied by satellite-retrieved $PM_{2.5}$ concentrations developed by Ma et al.[30] to calculate the ambient $PM_{2.5}$ concentrations for all scenarios.

Similar methods have been widely adopted in earlier studies to quantify the impact of polices addressing climate change [31,32] and other assessments like trade-driven air quality changes [33]. We should note that though it is not perfect, such methodology offers an effective means for the purpose of our analysis.

2.3 Model evaluation and uncertainties

The WRF-Chem model applied in this study has been widely used for modeling air quality of different regions across the world including East Asia, and detailed model performance has been extensively evaluated against both satellite retrievals and ground-based observations [29,34,35].

Though we mainly focus on the change ratios between various scenarios and 2015, it is necessary to evaluate the ability of our model to capture the spatial distribution and seasonal variation of $PM_{2.5}$ concentration. Ground-based observations at almost 1500 stations from the national air quality monitoring network were used to compare with the WRF-Chem simulations in 2015, the base run. The observational data are archived at air monitoring data center of Ministry of Ecology and Environment of the People's Republic of China (http://datacenter.mep.gov.cn). As shown in Supplementary Fig. 10, the simulated $PM_{2.5}$ concentration enjoys a relatively high Pearson correlation coefficient (R) of ~0.81 with the ground-based observations, while the mean bias is about 4.11 μg/m^3. And spatial distribution of annual mean $PM_{2.5}$ simulation is shown in

Supplementary Fig.12, overlaid with annual mean in-situ surface observations in 2015. The model simulation shows consistent spatial distribution characteristics of station observations, also with the satellite derived $PM_{2.5}$ product from Ma et al.[36] (Supplementary Fig. 12). The WRF-Chem simulation would overestimate in some areas, like Sichuan Basin, but still within a reasonable range. Also, the seasonal variation has also been well captured (Supplementary Fig. 13).

In addition, the ground-level satellite-retrieved $PM_{2.5}$ estimates we applied are highly consistent (R^2=0.75, slope=0.91) with $PM_{2.5}$ concentrations from in-situ surface monitors (the Figure 2 in Ma et al.[30]). The satellite-derived $PM_{2.5}$ concentration dataset has been applied broadly in the literature to represent the spatiotemporal distribution of $PM_{2.5}$ exposure in China [38,39].

Supplementary Note 3: Special case caused by ammonia emission

3.1 Agriculture and ammonia emissions in future scenarios

In China, both livestock and fertilizer application play significant roles[40,41], respectively accounting for approximately 54% and 33% of the total ammonia emissions. So, agricultural activity levels and emission factors related to both livestock and planting are the keys of projecting future NH_3 emissions in China.

(I) Agriculture in the future scenarios

Agriculture in future SSP-RCP scenarios is jointly determined by the socio-economic assumptions in the SSP baselines and the climate targets in the RCPs.

The demands for agricultural products and energy services differ across the SSPs (Supplementary Table 13), resulting from huge differences in socio-economic development pathways, population, consumption habits and diets [4]. On the other hand, the climate mitigation policies (i.e. RCPs) imposed on the SSPs would also profoundly affect the structure and volume of agricultural demands, through interactions with bioenergy and afforestation under policy assumptions and radiative forcing targets for GHGs emissions [6].

In GCAM, historical agricultural production and consumption of crops and livestock from the Food and Agriculture Organization (FAO) are used for calibration [9]. As for projections in scenarios, livestock production is closely related to food demand, while crop productions have complex interactions with bioenergy and afforestation. Here are detailed explanations for livestock husbandry and agricultural planting.

i) Livestock production

In GCAM, per capita food demand is estimated using the evolution of demand relationships across large changes in income and prices, based on the approach documented in Edmonds et al. (2017) . As shown in Supplementary Table 13, with the

implementation of climate policy (i.e., RCP2.6 and RCP1.9), food demand decreases relative to the according REF scenario for the same period, mainly due to increased food prices and limited food waste [4,9]. Pressures to expand forests and bioenergy under climate polices would drive up food prices, with several folds increase in producer prices for agricultural commodities [9]. These increases in food prices lead to declines in food consumption, a phenomenon observed earlier by Wise et al. (2009), which has been embedded in food demand estimation.

As GCAM operates by determining a set of prices that ensure supply is equal to demand for all time steps, the total food demand would determine the total food supply. Food supply would affect the pattern of agricultural production, and in turn affect related emissions. For livestock, GCAM uses logit sharing approach to make economic choices [44,45], and calculate the shares for different production systems and feed sources. Assumed logit parameters are used to dictate substitution between different commodities.

ii) Agricultural planting

Aside from food demand, crop production in the climate mitigation scenarios also has complex interactions with bioenergy and afforestation.

As shown in Supplementary Figure 2, the climate policies (i.e. RCPs) would drive up the demand for bioenergy [9,46]. Cropland used to grow energy crops increases significantly compared to the SSPs baseline scenarios (i.e. SSPx-REF) (Supplementary Table 14), which could drive up food prices and then reduce food demand as mentioned above. The expanding cultivation of energy crops could encourage use of nitrogenous fertilizers and increase NH_3 emission, which is also particularly relevant to emission factors.

(II) NH_3 emission factors in the future scenarios

GCAM include both NH_3 emissions from agricultural practices, agricultural waste

burning and other sectors. For livestock, key sources of ammonia emissions are in the various steps of the livestock production and animal manure management chains [40].

In general, agricultural non-CO_2 emissions in the future will depend on changes in drivers (i.e., agricultural production) and any reduction in emissions factors induced by either the carbon price via a MAC curve (Supplementary Note 1.4) or air pollution policies (Supplementary Note 1.5). It mainly includes substitution of urea fertilizers, rapid incorporation of solid manure, low nitrogen feed, bio-filtration and so on. Besides, some mitigation measures are not explicitly included in the depicted air pollution control, though they may be implicitly included in the MAC curves, like increasing nitrogen use efficiency.

(III) Increased NH_3 emission in the future scenarios

Above all, NH_3 emission in the future will depend on changes in drivers (i.e., agricultural activities) and any reduction in emissions factors induced by either the carbon price (via a MAC curve) or air pollution policies. Agricultural activity levels are closely related to the food, feed and bioenergy demand, from the final solution of SSP-RCP in GCAM model. The projected agricultural emission factors in China are jointly decided by both the baseline regional emission factors dataset and the air pollution control assumption in the SSPs.

The increase of NH_3 in our results is mainly due to the increase utilization of bioenergy, as for air pollution control policies are only implemented in the SSPs (i.e., SSPx-RCPs/REF share the same). Huge rises exist in agricultural production of energy crops, and residue and wastes for energy in all 2.6 W/m^2 (i.e. RCP2.6) and 1.9 W/m^2 (i.e. RCP1.9) scenarios (Supplementary Table 14).

Increasing nitrogen use efficiency of fertilizer application could contribute to reductions in both NH_3 and N_2O emissions [47,48]. Our results also suggest the importance of nitrogen management in agriculture.

3.2 Impacts of ammonia emission change

The $PM_{2.5}$ concentration of the SSP1_RCP2.6 scenario is slightly higher than that of SSP1_REF in 2030, as shown in Supplementary Fig. 8a. The positive deviations are concentrated eastern and southern China, especially in provinces Henan, Hubei and Hunan, with an approximate scope of 0-2 μg/m^3. which is mainly caused by the higher ammonia emissions.

As the model configuration are kept definitely same for all scenarios, the total emissions for various scenarios from GCAM is the only variable. According to Fig. 2 and Supplementary Table 15, annual total emissions of carbon and other pollutants are reduced in SSP1_RCP2.6 scenario from SSP1_REF_2030, except ammonia (NH_3). The increased NH_3 emission is mainly from land use sector, with higher production of energy crops for biomass fuels, under efforts to mitigate [9]. As shown in Supplementary Fig. 8b, the NH_3 emission in SSP1_RCP2.6_2030 is higher than that in SSP1_REF_2030 in eastern China, provinces with large populations and agricultural outputs like Henan, Shandong and Jiangsu [49]. The spatial pattern is similar to the difference of simulated $PM_{2.5}$ annual mean concentration between the two scenarios (Supplementary Fig. 8a).

Ammonia is an important precursor for the formation of secondary inorganic particles in the atmosphere. After being discharged into the atmosphere, ammonia gas reacts with sulfuric acid generated by SO_2 and nitric acid generated by NOx, and then produce secondary inorganic particles such as ammonium sulfate and ammonium nitrate. Ammonia emission contributes about 29.8% of the annual mean concentration of $PM_{2.5}$ in China, especially nitrate and ammonium with a high contribution rate of 99.5% while only 4.2% for sulfate [50]. Previous research has found that emissions of NH_3 contribute to nearly 30% of China's annual average concentration of $PM_{2.5}$, and an increasing NH_3 emission would hinder $PM_{2.5}$ mitigation by enhancing ammonium nitrate formation, a key species of secondary $PM_{2.5}$ [51].

The Primary emission of $PM_{2.5}$ components in the SSP1_RCP2.6_2030 scenario is lower than the SSP1_REF_2030 scenario, as demonstrated by black carbon in Supplementary Fig. 14a. In contrast, the nitrate and ammonium are about respectively 0.2~1 μg /m^3 and 0.2~0.6 μg/m^3 higher than that in the SSP1_REF_2030 scenario (Supplementary Fig. 14). In addition, the spatial distributions of the differences in nitrate and ammonium is highly consistent with the difference in ammonia emissions (Supplementary Fig. 8). The higher annual average $PM_{2.5}$ concentration in scenario SSP1_RCP2.6_2030 can be considered as the contribution of increase in secondary particulate matter caused by increased ammonia emissions. Here, the co-benefits of carbon mitigation on air pollution is offset by enhanced secondary $PM_{2.5}$ due to increased ammonia emissions.

3.3 Evaluation of scenarios employing measures to control the NH_3

To explore the potential of employing various additional measures to control NH_3 for air quality improvement, additional air quality modeling experiments of "reduced NH_3 emissions" have been designed.

We choose the scenarios of SSP1_RCP2.6 and SSP1_RCP1.9 in 2030 to evaluate the potential of employing various additional measures to control NH_3 (Supplementary Table 16), since SSP1 is the pathways which could bring great environmental and health benefits for China at an affordable cost in the long-run.

As ammonia emission rate decreases in the SSP1_RCP2.6_cutNH_3 scenario, the $PM_{2.5}$ concentration declines a lot (Supplementary Fig. 15). As shown in Supplementary Table 17, in 2030, the $PM_{2.5}$ concentration can decrease to 30.09 μg/m^3 with 50% reduction in NH_3 under SSP1_RCP2.6 scenario, and to 29.71 μg/m^3 under SSP1_RCP1.9 scenario. We can briefly draw a conclusion that, when implementing climate policies, agricultural nitrogen management and ammonia emission control measures could bring great benefits. Only through policies that coordinated control of air pollution and carbon emissions can policymakers maximize the total benefits for society.

Supplementary Note 4: Decomposition of changes in $PM_{2.5}$-related deaths

To understand the huge rise of the $PM_{2.5}$-induced health burden in scenarios from 2030 to 2050, here we calculated the contributions from four key factors that might lead to the elevated health burden estimation, i.e., population size, age structure, $PM_{2.5}$ exposure and mortality rate unrelated to ambient $PM_{2.5}$ exposure.

Methodology of the decomposition:

We adopted the "Standardization and decomposition of rates" decomposition method developed by Das Gupta [52] and added better estimates in grid-scale [53]. The decomposition algorithm tests the sensitivities from an accumulative change in the input parameters in assessing $PM_{2.5}$-related deaths.

The sequence of changing parameters can be described as:

1. the increase in population size (population growth, denoted as "*PG*"),
2. the change in age structure (population aging, denoted as "*PA*"),
3. the change in $PM_{2.5}$ exposure (denoted as "*EXP*"),
4. the change in the rate of mortality which is unrelated to ambient $PM_{2.5}$ exposure (i.e., due to changes in access to care, treatment and other risk factors, denoted as "ORF)" calculations for each step of the changed factor illustrated below:

$$M_a = \sum_{i=1}^{80+} P_{a,i} \times I_{a,i} \times \frac{(RR_{a,i} - 1)}{PWRR_{a,i}}$$

$$M_{PG} = \sum_{i=1}^{80+} P_{b,i} \times I_{a,i} \times \frac{(RR_{a,i} - 1)}{PWRR_{a,i}}$$

$$M_{PA} = \sum_{i=1}^{80+} P_{b,i} \times I_{a,i} \times \frac{(RR_{a,i} - 1)}{PWRR_{a,i}}$$

$$M_{EXP} = \sum_{i=1}^{80+} P_{b,i} \times I_{a,i} \times \frac{(RR_{b,i} - 1)}{PWRR_{a,i}}$$

$$M_{ORF} = \sum_{i=1}^{80+} P_{b,i} \times I_{b,i} \times \frac{(RR_{b,i} - 1)}{PWRR_{b,i}} = M_b$$

Where suffix *a* and *b* represent the start and the end year of the decomposition period, *i* represents different age groups, *P* is the population size; *I* is the sum of national

baseline mortality rate for non-communicable diseases and lower respiratory infections; *RR* and *PWRR* stand for the relative risk and population-weighted relative risk, respectively. The difference between every consecutive step is an estimate of the contribution of each factor:

$$Effect_{PG} = (M_{PG} - M_a)/M_a \times 100\%$$

$$Effect_{PA} = (M_{PA} - M_{PG})/M_a \times 100\%$$

$$Effect_{EXP} = (M_{EXP} - M_{PA})/M_a \times 100\%$$

$$Effect_{ORF} = (M_{ORF} - M_{EXP})/M_a \times 100\%$$

As shown in Supplementary Fig. 16, population aging is the leading factor of the huge rises in health burden from 2030 to 2050 in the scenarios. Indeed, obvious trend of population aging shows from 2015 to 2030 to 2050 in the SSPs (Supplementary Fig. 17). And the elderly are always faced with greater health risk even at the same $PM_{2.5}$ exposure level [54]. And owing to the consistent mortality rate with that in 2015 as assumed in this study, the contribution of the mortality rate is 0% here.

Besides, both the shrinking population and the improved air quality contribute to alleviation in the $PM_{2.5}$ health burden to some extent, though it is not comparable to the impact of aging. The reductions in $PM_{2.5}$ exposure in the two RCPs from REF are not great enough to offset the increased $PM_{2.5}$-related deaths caused by population aging and to shake the huge death base. Thus, even with the 1.5°C or 2°C target, the rise of $PM_{2.5}$ related mortality could not be curbed by climate policies only.

Supplementary Note 5: Uncertainty analysis

5.1 The impact of various relative risk (RR) function on $PM_{2.5}$ related mortality

i) GEMM

In this study, we apply the GEMM to estimate $PM_{2.5}$-related mortality. We estimated the $PM_{2.5}$-related mortality in China for each scenario and year, by summing the gridded deaths calculated based on mean level RR derived from GEMM and the gridded $PM_{2.5}$ concentration. The uncertain intervals (i.e. error bars) were obtained by using the ratios of the confidence intervals (CIs) of national $PM_{2.5}$-related mortality at the national population-weighted $PM_{2.5}$ concentration level to constraint the summed value.

The 95% CIs of RR in GEMM were calculated by inserting (1.96 * the standard error of $\theta_{i,j}$, $SE(\theta_{i,j})$) into the functions as follows:

$$95\,\%\ CI\ of\ RR_{i,j,g} = exp\{\frac{(\theta_{i,j} \pm 1.96 \times SE(\theta_{i,j})) \times (log(\frac{C_g - C_0}{\alpha_{i,j}} + 1))}{1 + exp\{-\frac{C_g - C_0 - \mu_{i,j}}{v_{i.j}}\}}\}$$

Where, i and j stand for disease and age group; $\theta, \alpha, \ \mu\ \ v$ are fitted parameters of the concentration response functions for a given disease provided by Burnett et al.[54].

ii) IER

We also use the integrated exposure-response (IER) functions to assess the health impacts of $PM_{2.5}$ [55,56]. The IER functions were built to provide global estimates of relative risk (RR) of mortality over a wide range of ambient annual mean $PM_{2.5}$ concentrations for each health endpoint by using risk estimates from studies of ambient air pollution, household air pollution, and second-hand smoke exposure and active smoking. The IER functions present a nonlinear relationship between $PM_{2.5}$ concentrations and RR values, which have been deemed appropriate for the Chinese context [38,57,58]. According to the IER functions, five health endpoints have been shown

to be associated with $PM_{2.5}$ exposure: chronic obstructive pulmonary disease (COPD), ischemic heart disease (IHD), lung cancer (LC), acute lower respiratory infections (ALRI), and stroke. For each health endpoint, the attributable mortality caused by $PM_{2.5}$ exposure can be calculated as:

$$M_{g,j} = P_{g,j} \times I_i \times \frac{RR_{g,j} - 1}{RR_{g,j}}, where\ \overline{RR_{g,j}} = \frac{\sum P_{g,j} \times RR_{g,j}}{\sum P_{g,j}}$$

Where g and j stand for grid cell and age group; M is the number of deaths attributable to $PM_{2.5}$ exposure; P is the population; I is the national incidence rate for an endpoint; RR is the relative risk. RR is derived from the IER functions:

$$RR_{g,i,j}(C_g) = \begin{cases} 1 + \alpha(1 - e^{-\gamma(C_{i,j,g} - C_0)^{\delta}}) & if\ C_g > C_0 \\ 1 & otherwise \end{cases}$$

Where $RR_{i,j}$ is the RR of health endpoint i and age group j of a given annual average $PM_{2.5}$ concentration C in grid cell g (μg/m^3); C_0 is the theoretical minimum risk exposure level, which was set to 2.4-5.9 μg/m^3 according to GBD 2017 study [59]; α, γ and δ are parameters of the IER functions for each health endpoint.

We use a set of 1,000 IER parameters (n=1000) for the five health endpoints and age stratum provided by Cohen et al.[56] to estimate uncertainties. We defined the 95% confidence intervals from the 2.5 and 97.5 percentiles of the RR probability distribution, and the mean values of RR were used to represent our results.

To compare with the results of IER, we use GEMM to calculate the mortality attribute to $PM_{2.5}$ of five health endpoints including COPD, IHD, LC, ALRI and stroke [54]. The results of different methods are in Supplementary Fig. 18 and Fig. 19. Moreover, as shown in Supplementary Table 18, health benefit could still largely offset the mitigation costs.

5.2 The impact of the value of statistical life (VSL)

Scaling approach is one of the three types of VSL estimation methods (direct estimation, meta-analysis, and scaling) and has been widely used in developing countries without comprehensive VSL research. The scaling approach uses VSL from other developed countries (such as the United States) or from certain domestic cities where VSL studies have been conducted, and then calibrates the VSL based on income differences.

In this study, we calculate VSL for 2015 and future years using scaling approach based on studies in Chinese city. The future per capita disposable income (INC) is extrapolated based on GDP growth rate with the assumption that GDP growth equals to INC growth in the future, due to the GDP growth was comparable with INC growth according to Chinese official statistical data. The predicted GDP is from SSP Database (https://tntcat.iiasa.ac.at/SspDb/dsd?Action=htmlpage&page=about). And we adopt contingent valuation conducted in Chongqing which presented a linear relationship between VSL and income (i.e., VSL increased by 14,550 USD with annual income increases of 145.8 USD) [60,61]. Supplementary Table 19 shows the values of $VSL_{baseline}$ and their sources.

In addition, we adopt another method to estimate VSL according to guidance of US EPA [66,67]:

$$VSL_{target}=VSL_{Base} \times (\frac{Y_{target}}{Y_{Base}})^{\beta}$$

Where, VSL_{target} is the VSL for target year in china; VSL_{Base} is a baseline VSL from previous studies, where we use the value from Organization for Economic Co-operation and Development (OECD) in 2005 and the value is 3million dollars; Y_{target} and Y_{Base} are economic indicators which could be gross national income (GNI) per capita, GDP per capita and INC, in this study, we use GDP; β is the economic elasticity and we choose 0.4 and 0.8.

Supplementary Figure 20 presents different VSL data based on various baseline studies and various coefficients. We find that when using the OECD baseline VSL, the

estimated VSL in China were almost one order of magnitude higher than the results using the data from local VSL studies, and the impact of the baseline VSL was far bigger than the influence caused by the economic elasticity coefficient. The estimated VSL in this study is on the same order of magnitude as Liang et al.[67]. Overall, moderate VSLs were estimated based on the calculation method used in this study.

5.3 Implicit cost savings in air pollution by climate polices.

The decarbonized energy system under climate policies would indeed bring co-reductions in air pollutant emissions, thus induce significant cost savings for air pollution control, or in other words, avoided expenses for air pollution control. It could be an additional co-benefit of climate policies, which has not been covered in our cost-benefit analysis.

To assess this part of co-benefit, we estimate the annual avoided abatement cost for air pollution control in each scenario. It was calculated based on the air pollutant reduction ratios from the 2010 level (Supplementary Table 20) and the marginal abatement cost curves from Zhang et al.[68], which describe the relationship between marginal cost and reduction ratio. The curves were retrieved from a linear programming algorithm model, International Control Cost Estimate Tool, and updated with cost data for applications of 56 types of end-of-pipe technologies and five types of renewable energy in 10 major sectors [68]. Both the end-of-pipe measures and energy structure adjustment were applied in the development of the marginal abatement cost curves.

The annual avoided abatement costs for air pollution control in each scenario are shown in Supplementary Fig. 21, using a discount rate of 5%. The abatement cost reduction for VOCs from 2010 level is rather significant in the climate policy scenarios, which is mainly contributed by the particularly expensive marginal abatement costs as estimated. In addition, the avoided air pollution control costs for primary PM are not estimated here, since primary PM are not included in projected emissions by GCAM,

excluding BC and OC which, however, lack corresponding cost estimates. What's more, according to the bottom-up marginal cost curve in Zhang et al. [68], the magnitude of the primary PM cost curve is at the same order with SO_2, much smaller than other 3 pollutants. Thus, the avoided cost here would be underestimated to some extent in this aspect.

Then, the annual cost savings in air pollution control by climate mitigation were estimated by comparing RCPs scenarios with the corresponding REF (Supplementary Table 21). This part of cost savings brought by 1.5℃ climate policy can be 14.4 and 150.3 billion US$ in 2030 and 2050, relative to REF respectively. The climate mitigation scenarios (i.e., RCP2.6 and RCP1.9) could bring larger co-reductions in air pollutant emissions with deeper decarbonized energy systems [69,70], thus induce larger cost reductions than the REF case.

We have compared the annual cost results with those in existing studies [71], and found them all in the order of hundreds billion US dollars, for example mainly in 100~300 billion US$ in 2050. Still, great uncertainties lie in the development of technologies and the estimated cost. In addition, recent studies showed that the benefits of end-of-pipe control reductions are mostly exhausted by 2030 in China [69]. Since this article mainly focuses on the health benefits of coordinated reduction of air pollutants under climate policies, rough estimation has been conducted here without further discussion about factors and uncertainties.

References

1 Kriegler, E. et al. The need for and use of socio-economic scenarios for climate change analysis: A new approach based on shared socio-economic pathways. Global Environmental Change **22**, 807-822, doi:https://doi.org/10.1016/j.gloenvcha.2012.05.005 (2012).

2 van Vuuren, D. P. et al. The representative concentration pathways: an overview. CLIMATIC CHANGE **109**, 5, doi:10.1007/s10584-011-0148-z (2011).

3 O'Neill, B. C. et al. A new scenario framework for climate change research: the concept of shared socioeconomic pathways. CLIMATIC CHANGE **122**, 387-400, doi:10.1007/s10584-013-0905-2 (2014).

4 Riahi, K. et al. The Shared Socioeconomic Pathways and their energy, land use, and greenhouse gas emissions implications: An overview. Global Environmental Change **42**, 153-168, doi:https://doi.org/10.1016/j.gloenvcha.2016.05.009 (2017).

5 O'Neill, B. C. et al. The roads ahead: Narratives for shared socioeconomic pathways describing world futures in the 21st century. Global Environmental Change **42**, 169-180, doi:https://doi.org/10.1016/j.gloenvcha.2015.01.004 (2017).

6 Kriegler, E. et al. A new scenario framework for climate change research: the concept of shared climate policy assumptions. CLIMATIC CHANGE **122**, 401-414, doi:10.1007/s10584-013-0971-5 (2014).

7 Kriegler, E. et al. Fossil-fueled development (SSP5): An energy and resource intensive scenario for the 21st century. Global Environmental Change **42**, 297-315, doi:https://doi.org/10.1016/j.gloenvcha.2016.05.015 (2017).

8 Rogelj, J. et al. Scenarios towards limiting global mean temperature increase below 1.5 °C. Nature Climate Change **8**, 325-332, doi:10.1038/s41558-018-0091-3 (2018).

9 Calvin, K. et al. The SSP4: A world of deepening inequality. Global Environmental Change **42**, 284-296, doi:https://doi.org/10.1016/j.gloenvcha.2016.06.010 (2017).

10 Hoesly, R. M. et al. Historical (1750–2014) anthropogenic emissions of reactive gases and aerosols from the Community Emissions Data System (CEDS). Geosci. Model Dev. **11**, 369-408, doi:10.5194/gmd-11-369-2018 (2018).

11 USEPA. Global Non-CO2 Greenhouse Gas Emission Projection & Mitigation Potential Report. . (United States Environmental Protection Agency, Office of Atmospheric Programs. , 2019).

12 Amann, M. et al. Cost-effective control of air quality and greenhouse gases in Europe: Modeling and policy applications. Environmental Modelling & Software **26**, 1489-1501, doi:https://doi.org/10.1016/j.envsoft.2011.07.012 (2011).

13 Amann, M., Klimont, Z. & Wagner, F. Regional and Global Emissions of Air Pollutants: Recent Trends and Future Scenarios. Annual Review of Environment and Resources **38**, 31-55, doi:10.1146/annurev-environ-052912-173303 (2013).

14 Rao, S. et al. Future air pollution in the Shared Socio-economic Pathways. Global Environmental Change **42**, 346-358, doi:https://doi.org/10.1016/j.gloenvcha.2016.05.012 (2017).

15 Grell, G. A. et al. Fully coupled "online" chemistry within the WRF model. Atmos. Environ. **39**, 6957-6975, doi:10.1016/j.atmosenv.2005.04.027 (2005).

16 Xin Huang, Y. S., Chun Zhao3. Pathways of sulfate enhancement by natural and anthropogenic

mineral aerosols in China. J GEOPHYS RES-ATMOS, doi:10.1002/ (2014).
17 Lo, J. C. F., Yang, Z. L. & Pielke, R. A. Assessment of three dynamical climate downscaling methods using the Weather Research and Forecasting (WRF) model. J. Geophys. Res. Atmos. **113**, D09112, doi:10.1029/2007jd009216 (2008).
18 Berge, E., Huang, H. C., Chang, J. & Liu, T. H. A study of the importance of initial conditions for photochemical oxidant modeling. J. Geophys. Res. Atmos. **106**, 1347-1363, doi:10.1029/2000jd900227 (2001).
19 Iacono, M. J. et al. Radiative forcing by long-lived greenhouse gases: Calculations with the AER radiative transfer models. J GEOPHYS RES **113**, doi:10.1029/2008jd009944 (2008).
20 Ek, M. B. et al. Implementation of Noah land surface model advances in the National Centers for Environmental Prediction operational mesoscale Eta model. J. Geophys. Res. Atmos. **108(D22)**, 8851, doi:10.1029/2002jd003296 (2003).
21 Hong, S.-Y., Noh, Y. & Dudhia, J. A New Vertical Diffusion Package with an Explicit Treatment of Entrainment Processes. Monthly Weather Review **134**, 2318-2341, doi:10.1175/mwr3199.1 (2006).
22 Lin, Y. L., Farley, R. D. & Orville, H. D. Bulk Parameterization Of The Snow Field In A Cloud Model. Journal of Climate and Applied Meteorology **22**, 1065-1092, doi:10.1175/1520-0450(1983)022<1065:Bpotsf>2.0.Co;2 (1983).
23 Grell, G. A. & Freitas, S. R. A scale and aerosol aware stochastic convective parameterization for weather and air quality modeling. Atmos. Chem. Phys. **14**, 5233-5250, doi:10.5194/acp-14-5233-2014 (2014).
24 Li, M. et al. MIX: a mosaic Asian anthropogenic emission inventory under the international collaboration framework of the MICS-Asia and HTAP. Atmos. Chem. Phys. **17**, 935-963, doi:10.5194/acp-17-935-2017 (2017).
25 Guenther, A. B. et al. The Model of Emissions of Gases and Aerosols from Nature version 2.1 (MEGAN2.1): an extended and updated framework for modeling biogenic emissions. Geosci. Model Dev. **5**, 1471-1492, doi:https://doi.org/10.5194/gmd-5-1471-2012 (2012).
26 Bell, M. L., Morgenstern, R. D. & Harrington, W. Quantifying the human health benefits of air pollution policies: Review of recent studies and new directions in accountability research. Environmental Science & Policy **14**, 357-368, doi:https://doi.org/10.1016/j.envsci.2011.02.006 (2011).
27 Giani, P. et al. Short-term and long-term health impacts of air pollution reductions from COVID-19 lockdowns in China and Europe: a modelling study. The Lancet Planetary Health **4**, e474-e482, doi:https://doi.org/10.1016/S2542-5196(20)30224-2 (2020).
28 Venter, Z. S., Aunan, K., Chowdhury, S. & Lelieveld, J. COVID-19 lockdowns cause global air pollution declines. PNAS, 202006853, doi:10.1073/pnas.2006853117 (2020).
29 Huang, X. et al. Enhanced secondary pollution offset reduction of primary emissions during COVID-19 lockdown in China. National Science Review, doi:10.1093/nsr/nwaa137 (2020).
30 Ma, Y. et al. A study on the short-term impact of fine particulate matter pollution on the incidence of cardiovascular diseases in Beijing, China. ATMOS ENVIRON **215**, 116889, doi:10.1016/j.atmosenv.2019.116889 (2019).
31 West, J. J. et al. Co-benefits of mitigating global greenhouse gas emissions for future air quality and human health. Nature Climate Change **3**, 885-889, doi:10.1038/nclimate2009 (2013).

32 Shindell, D. T., Lee, Y. & Faluvegi, G. Climate and health impacts of US emissions reductions consistent with 2 °C. Nature Climate Change **6**, 503-507, doi:10.1038/nclimate2935 (2016).
33 Wang, H. et al. Trade-driven relocation of air pollution and health impacts in China. NAT COMMUN **8**, doi:10.1038/s41467-017-00918-5 (2017).
34 Huang, X. et al. Effects of aerosol–radiation interaction on precipitation during biomass-burning season in East China. Atmos. Chem. Phys. **16**, 10063-10082, doi:10.5194/acp-16-10063-2016 (2016).
35 Tang, R., Huang, X., Zhou, D. & Ding, A. Biomass-burning-induced surface darkening and its impact on regional meteorology in eastern China. Atmos. Chem. Phys. **20**, 6177-6191, doi:10.5194/acp-20-6177-2020 (2020).
36 Ma, Z. W. et al. Satellite-Based Spatiotemporal Trends in PM2.5 Concentrations: China, 2004–2013. ENVIRON HEALTH PERSP **124**, 184-192, doi:10.1289/ehp.1409481 (2016).
37 Ma, Z. W., Liu, R. Y., Liu, Y. & Bi, J. Effects of air pollution control policies on PM2.5 pollution improvement in China from 2005 to 2017: a satellite-based perspective. ATMOS CHEM PHYS **19**, 6861-6877, doi:10.5194/acp-19-6861-2019 (2019).
38 Liu, M. et al. Spatial and temporal trends in the mortality burden of air pollution in China: 2004–2012. Environment International **98**, 75-81, doi:https://doi.org/10.1016/j.envint.2016.10.003 (2017).
39 Wang, Q., Wang, J., He, M. Z., Kinney, P. L. & Li, T. A county-level estimate of PM2.5 related chronic mortality risk in China based on multi-model exposure data. Environment International **110**, 105-112, doi:https://doi.org/10.1016/j.envint.2017.10.015 (2018).
40 Huang, X. et al. A high-resolution ammonia emission inventory in China. GLOBAL BIOGEOCHEM CY **26**, n/a-n/a, doi:10.1029/2011gb004161 (2012).
41 Ge, P., Chen, M., Cui, Y. & Nie, D. The Research Progress of the Influence of Agricultural Activities on Atmospheric Environment in Recent Ten Years: A Review. Atmosphere **12**, doi:10.3390/atmos12050635 (2021).
42 Edmonds, J. A., Link, R., Waldhoff, S. T. & Cui, R. A Global Food Demand Model For The Assessment Of Complex Human-Earth Systems. Climate Change Economics **08**, 1750012, doi:10.1142/S2010007817500129 (2017).
43 Wise, M. et al. Implications of Limiting CO2 Concentrations for Land Use and Energy. Science **324**, 1183-1186, doi:10.1126/science.1168475 (2009).
44 Mcfadden, D. Conditional logit analysis of qualitative choice behavior. Frontiers in Econometrics (1974).
45 Train, K. E. Discrete Choice Methods with Simulation. (Discrete Choice Methods With Simulation, 2009).
46 Doelman, J. C. et al. Exploring SSP land-use dynamics using the IMAGE model: Regional and gridded scenarios of land-use change and land-based climate change mitigation. Global Environmental Change **48**, 119-135, doi:https://doi.org/10.1016/j.gloenvcha.2017.11.014 (2018).
47 Gu, B., Sutton, M. A., Chang, S. X., Ge, Y. & Chang, J. Agricultural ammonia emissions contribute to China's urban air pollution. Frontiers in Ecology and the Environment **12**, 265-266, doi:10.1890/14.Wb.007 (2014).
48 Wang, X.-D. et al. Responses of greenhouse gas emissions to residue returning in China's croplands

and influential factors: A meta-analysis. Journal of Environmental Management **289**, 112486, doi:https://doi.org/10.1016/j.jenvman.2021.112486 (2021).
49 Ning, q. & Hu, l. China Agricultural statistical yearbook 2018. (China Agricultural Press, 2018).
50 Xue, W. B., Xu, Y. L., Tang, X. L., Lei, Y. & Wang, J. N. Impacts of ammonia emission on PM2.5 pollution in China. China Environmental ence **36**, 3531-3539 (2016).
51 Liu, M. et al. Ammonia emission control in China would mitigate haze pollution and nitrogen deposition, but worsen acid rain. PNAS **116**, 7760, doi:10.1073/pnas.1814880116 (2019).
52 Das Gupta, P. Standardization and decomposition of rates: a user's manual. (U.S. Department of Commerce, Economics and Statistics Administration, Bureau of the Census, 1993).
53 Apte, J. S., Marshall, J. D., Cohen, A. J. & Brauer, M. Addressing Global Mortality from Ambient PM2.5. Environmental Science and Technology **49**, 8057-8066, doi:10.1021/acs.est.5b01236 (2015).
54 Burnett, R. et al. Global estimates of mortality associated with long-term exposure to outdoor fine particulate matter. PNAS **115**, 9592-9597, doi:10.1073/pnas.1803222115 (2018).
55 Burnett, R. T. et al. An Integrated Risk Function for Estimating the Global Burden of Disease Attributable to Ambient Fine Particulate Matter Exposure. ENVIRON HEALTH PERSP **122**, 397-403, doi:10.1289/ehp.1307049 (2014).
56 Cohen, A. J. et al. Estimates and 25-year trends of the global burden of disease attributable to ambient air pollution: an analysis of data from the Global Burden of Diseases Study 2015. The Lancet **389**, 1907-1918, doi:10.1016/s0140-6736(17)30505-6 (2017).
57 Xie, R. et al. Long-term trend and spatial pattern of PM2.5 induced premature mortality in China. Environment International **97**, 180-186, doi:https://doi.org/10.1016/j.envint.2016.09.003 (2016).
58 Zheng, H. et al. Transition in source contributions of PM2.5 exposure and associated premature mortality in China during 2005–2015. Environment International **132**, 105111, doi:https://doi.org/10.1016/j.envint.2019.105111 (2019).
59 Stanaway, J. D. et al. Global, regional, and national comparative risk assessment of 84 behavioural, environmental and occupational, and metabolic risks or clusters of risks for 195 countries and territories, 1990–2017: a systematic analysis for the Global Burden of Disease Study 2017. The Lancet **392**, 1923-1994, doi:https://doi.org/10.1016/S0140-6736(18)32225-6 (2018).
60 Zhang, M., Song, Y., Cai, X. & Zhou, J. Economic assessment of the health effects related to particulate matter pollution in 111 Chinese cities by using economic burden of disease analysis. Journal of Environmental Management **88**, 947-954, doi:https://doi.org/10.1016/j.jenvman.2007.04.019 (2008).
61 Wang, H., Mullahy, J., Chen, D., Wang, L. & Peng, R. in the Third International Health Economic Association Conference.
62 Yang, Z., Liu, P. & Xu, X. Estimation of social value of statistical life using willingness-to-pay method in Nanjing, China. Accident Analysis & Prevention **95**, 308-316, doi:https://doi.org/10.1016/j.aap.2016.04.026 (2016).
63 Huang, D., Andersson, H. & Zhang, S. Willingness to pay to reduce health risks related to air quality: evidence from a choice experiment survey in Beijing. Journal of Environmental Planning and Management **61**, 2207-2229, doi:10.1080/09640568.2017.1389701 (2017).
64 Hammitt, J. K., Geng, F., Guo, X. & Nielsen, C. P. Valuing mortality risk in China: Comparing stated-

preference estimates from 2005 and 2016. Journal of Risk and Uncertainty **58**, 167-186, doi:10.1007/s11166-019-09305-5 (2019).

65 Jin, Y., Andersson, H. & Zhang, S. Do preferences to reduce health risks related to air pollution depend on illness type? Evidence from a choice experiment in Beijing, China. Journal of Environmental Economics and Management **103**, 102355, doi:https://doi.org/10.1016/j.jeem.2020.102355 (2020).

66 EPA, U. S. Guidelines for Preparing Economic Analyses. (National Center for Environmental Economics Office of Policy, U.S. Environmental Protection Agency, 2014).

67 Liang, X. et al. Air quality and health benefits from fleet electrification in China. Nature Sustainability **2**, 962-971, doi:10.1038/s41893-019-0398-8 (2019).

68 Zhang, F. et al. Estimation of abatement potentials and costs of air pollution emissions in China. Journal of Environmental Management **260**, 110069, doi:https://doi.org/10.1016/j.jenvman.2020.110069 (2020).

69 Cheng, J. et al. Pathways of China's PM2.5 air quality 2015–2060 in the context of carbon neutrality. National Science Review, doi:10.1093/nsr/nwab078 (2021).

70 Xing, J. et al. The quest for improved air quality may push China to continue its CO₂ reduction beyond the Paris Commitment. PNAS, 202013297, doi:10.1073/pnas.2013297117 (2020).

71 Li, N. et al. Air Quality Improvement Co-benefits of Low-Carbon Pathways toward Well Below the 2 °C Climate Target in China. ENVIRON SCI TECHNOL **53**, 5576-5584, doi:10.1021/acs.est.8b06948 (2019).
